# Small spheroids for head and neck cartilage tissue engineering

**DOI:** 10.1038/s41598-024-83847-w

**Published:** 2024-12-30

**Authors:** Sven Reutter, Johann Kern, Yvonne Jakob, Nicole Rotter, David Gvaramia

**Affiliations:** 1 Department of Otorhinolaryngology, Head and Neck Surgery, Medical Faculty Mannheim, University of Heidelberg, Mannheim, Germany; 2 Department of Otorhinolaryngology, Head and Neck Surgery, University Clinic Mannheim, University of Heidelberg, Mannheim, Germany

**Keywords:** Cartilage, Spheroid, Elastic cartilage, Microtissues, Head and neck, Tissue engineering, Biotechnology, Regenerative medicine, Tissue engineering

## Abstract

The demand for cartilage reconstruction in the head and neck region arises frequently due to trauma, malignancies, and hereditary diseases. Traditional tissue engineering produces cartilage from a small biopsy by combining biomaterials and expanded cells. However, this top-down approach is associated with several limitations, including the non-uniform distribution of cells, lack of physiological cell-cell and cell-matrix interactions, and compromised mechanical properties and tissue architecture. The capacity of cells to aggregate into microtissues enables an alternative bottom-up approach to producing cartilage with or without further scaffolding support. Here we explored the optimal conditions for obtaining small spheroids from head and neck cartilage tissues. We used chondrocytes (CCs) and chondroprogenitors (CPCs) isolated from auricular and nasoseptal cartilage to prepare spheroids using ultra-low attachment (ULA) plates or micromass cultures. Different cell densities were tested to estimate the minimal cell number required for optimal spheroid formation. Furthermore, we evaluated the influence of key chondrogenic cytokines, such as transforming growth factor (TGF)-β, connective tissue growth factor (CTGF), and insulin-like growth factor (IGF)-1, on spheroid morphology and the production of cartilage extracellular matrix (ECM) components. Spheroids expressing cartilage markers were formed with 2.5 × 10^4^ cells in a commercially available chondrogenic differentiation medium on ULA plates but not in conventional micromass cultures. Differences were seen in auricular and nasal spheroids with respect to growth patterns and response to cytokine composition. Auricular spheroids were larger and showed size increase in culture, whereas nasal aggregates tended to shrink. Cytokines differentially influenced spheroid growth, and ECM structure and composition. Under all tested conditions, both spheroid types generated one or more cartilage ECM components, including elastin, which was also found in nasal spheroids despite their hyaline origin. Our results suggest that spheroid cultures can offer a viable approach to generating mature cartilage tissue without a biomaterial scaffold. Furthermore, nasal CCs and CPCs can be used to generate elastic cartilage. The findings of the study provide technical insights toward the goal of obtaining cartilage microtissues that can be potentially used for reconstructive procedures of HNC cartilage defects.

## Introduction

The key organs in the head and neck region are predominantly composed of cartilage. The human auricle is shaped by elastic cartilage, whereas hyaline cartilage is the main structural component of the nose, trachea, and parts of the larynx. Given its poor regenerative capacity, the need for reconstructive treatment of cartilage tissue in the head and neck frequently arises due to trauma, tumors, and congenital malformations, among others. Currently, autologous reconstruction using costal cartilage is considered the gold standard^1^. However, this approach can result in donor site morbidity, including complications like pneumothorax, thoracic scoliosis, and atelectasis^2^. Furthermore, costal cartilage does not always match the mechanical consistency of the target tissue, as is the case with the highly elastic auricle.

Cell-based therapies combined with tissue engineering (TE) techniques have proven successful in clinical settings for treating articular cartilage defects^3^, and they hold promise for therapeutic applications in the head and neck region^4^. These approaches involve the cultivation of chondrogenic cells in three-dimensional (3D) cultures, either with or without additional structural scaffold support. The use of 3D cultures allows for better replication of cell-cell contacts and interactions with the microenvironment, mimicking the in vivo conditions and preventing chondrocytes from de-differentiating into fibroblast-like cells^5^.

However, generating cartilage constructs on a human scale requires a significant number of cells, which necessitates large-scale cell expansion in conventional monolayer cultures. This poses a major challenge in cartilage TE since chondrocytes tend to adopt a fibroblastic phenotype when proliferating in 2D monolayers^5^. Recent studies have shown that chondrocytes undergo substantial dedifferentiation after four passages on tissue culture plastic^6^. Nevertheless, it has been demonstrated that the expanded chondrocytes can regain their chondrogenic phenotype upon reintroduction into 3D cultures^7,8^, indicating that the fibroblastic phenotype acquired in monolayers may be transient and reversible. Therefore, extensive chondrocyte expansion for TE applications can be viable as long as an optimal 3D culture environment is established to facilitate renewed chondrogenic differentiation in the expanded cells.

Scaffold-free 3D cultures rely on cell aggregation and the self-assembly of multicellular spheroid structures, mimicking the process of mesenchymal condensation that naturally occurs during chondrogenesis in embryonic development^9,10^. Several commonly used scaffold-free culture methods for stimulating chondrogenesis include pellet, micromass (MM), and spheroid cultures. In pellet cultures, cells are aggregated using centrifugal force, while MM cultures involve placing a highly concentrated drop of a cell suspension in the interior of a well plate, allowing the cells to adhere and self-assemble^11^. Research has shown that when compared to pellet culture, mesenchymal MM cultures tend to produce a higher amount of cartilage-specific extracellular matrix (ECM), such as collagen type II (collagen II) while reducing the production of collagen type I (collagen I)^11^. Spheroid cultures, on the other hand, utilize ultra-low attachment (ULA) plates to promote cell aggregation. This method results in more consistent cell aggregation, as it reduces the variation in cell numbers that can occur during centrifugation. Similar to MM cultures, spheroid cultures are thus considered superior to standard pellet cultures^5^. However, it is notable that a direct comparison between MM and spheroid cultures in terms of chondrogenesis has yet to be conducted. Chondrogenic scaffold-free cultures are usually produced by aggregation of mesenchymal stromal cells (MSCs) or chondrocytes and their subsequent culture in a chondrogenic differentiation medium. Furthermore, chondrogenic progenitor cells (CPCs) may provide a viable alternative cell source. CPCs have demonstrated some superiority in chondrogenic potential as compared to chondrocytes and bone-marrow-derived MSCs, showing a lowered tendency towards the formation of fibrocartilage or chondrocyte hypertrophy^12,13^. Furthermore, CPCs exhibit increased telomerase activity and maintain higher expression levels of SOX-9 compared to fully differentiated chondrocytes, which enables them to have a higher proliferation capacity^14,15^. CPCs can be isolated by fibronectin adhesion assay, sorting by surface markers, or by cell outgrowth from cartilage explants^13^. The latter method takes advantage of the higher migratory capacity of CPCs in comparison to chondrocytes, resulting in a selective outgrowth of CPCs from the cultured cartilage explants^13,15^. Recent research demonstrated an improved chondrogenic capacity of hyaline cartilage CPCs isolated by explant outgrowth as compared to those isolated by fibronectin adhesion assay, suggesting that isolation by outgrowth might be a preferred method to obtain CPCs^13^.

Culture conditions play a crucial role in inducing in vitro chondrogenesis, whereby supplementation of culture medium with various factors, such as ascorbic acid, glucocorticoids, and cytokines, is necessary to promote cell proliferation and cartilage ECM production. Among the growth factors, Transforming Growth Factor β1 (TGF-β1) has a long history of use in chondrogenic cultures^16^. However, some studies have shown that another member of the TGF-β family, TGF-β3, is superior to TGF-β1 in stimulating chondrogenesis in vitro^17^. Connective Tissue Growth Factor (CTGF), which is involved in the TGF-β signaling cascade during wound healing, plays an important role in cartilage development and promotes elastin expression^18^, thus contributing to the formation of elastic cartilage. Insulin-like growth factor (IGF)-1 is another cytokine that stimulates elastin production in chondrocytes^19,20^. Overall, IGF-1 is considered the most important anabolic growth factor for cartilage, promoting collagen II production while inhibiting chondrocyte hypertrophy and apoptosis. These factors, either individually or in combination, stimulate various aspects of chondrogenesis, including cell proliferation and the production of cartilage-specific ECM. Therefore, they are suitable candidates for supplementation in scaffold-free 3D cultures, aiding in the development of an optimal environment for chondrogenic differentiation and tissue formation.

Scaffold-free cultures typically require a high cell density to promote self-assembly and generate mature functional constructs. In the case of cartilage MM and pellet cultures, cell numbers typically range from 1 × 10^5^ to 5 × 10^5^ cells per construct^21–24^. However, such high cell densities limit the feasibility of high-throughput analyses and necessitate extensive cell expansion through multiple passages in a conventional monolayer culture. Further drawbacks of large cartilage constructs are an inhomogeneous distribution of ECM and viable cells due to poor diffusion of nutrients and cytokines towards the center of cell aggregates^25–27^. Recent studies have demonstrated that the limitations associated with nutrient diffusion can be reduced in smaller spheroids, demonstrating superior maturation capacity, viability, and improved ECM distribution^28^. These microspheroids can then be employed as modular building blocks for producing TE cartilage either by further fusion or embedding into biomaterials^28–31^.

Here we aimed to produce scaffold-free chondrogenic cultures of reduced size without compromising the production of cartilage ECM components. To achieve this, we explored the potential for assembly of head and neck cartilage spheroids (HNC spheroids) using minimal numbers of nasal and auricular chondrocytes and CPCs, ranging from 0.5 to 10 × 10^4^ cells per construct. We deliberately expanded the cells in monolayers to evaluate their capacity for re-differentiation in spheroids. As a reference, we compared the small spheroids with MM cultures containing similar cell densities. To promote construct maturation and the production of cartilage ECM, we supplemented the spheroid cultures with growth factors such as TGF-β1, TGF-β3, IGF-1, or CTGF. Specifically, we focused on elastin, which is characteristic of elastic cartilage found in auricular tissue. Our investigation aimed to determine whether spheroid cultures composed of cells from hyaline nasal septal cartilage, which do not naturally express elastin, could be suitable for the production of elastic cartilage in vitro. Consequently, we evaluated the potential of an IGF-1 and CTGF combination to induce elastin production in hyaline cartilage spheroids.

## Methods

### Isolation and culture of chondrocytes (CCs) and chondrogenic progenitor cells (CPCs)

The isolation of human nasal and auricular chondrocytes from healthy donors was approved by the Ethics Committee of the Medical Faculty of Mannheim (2018–507 N-MA, 2018–584 N-MA). All methods in the study were performed in accordance with the relevant guidelines and regulations. Cartilage material was obtained following informed consent from healthy donors undergoing reconstructive surgeries at the Department of Otorhinolaryngology, Head and Neck Surgery, University Hospital Mannheim, Germany. Patient information, including age and gender, was anonymized and made inaccessible in compliance with the ethics approvals. The perichondrium was removed from cartilage tissue (ear or nose) which was then minced into small pieces (< 1 mm^3^) and subsequently digested overnight at 37 °C in 0.1% Collagenase II (ThermoFisher Scientific) in DMEM/F12 (1:1) + GlutaMAXTM-I supplemented with 10% FCS and 0.05 mg/mL Gentamicin (from here onwards, standard medium). Next, cells were filtered through a 100 μm cell strainer, washed, counted, and seeded in a standard medium. The cells were cultured for further expansion until passage (P)4 in a standard medium.

CPCs were derived by the primary explant technique, as previously described^15^. Briefly, donor cartilage tissues were washed in FCS-free DMEM/F12 medium and cut into pieces of approximately 1 mm^3^ after careful removal of fat tissue and perichondrium. Cartilage pieces were plated with standard tissue culture flasks and the explants were allowed to attach to the plastic for a few minutes before covering them with medium. The adhesion of the explants was allowed for at least 24–48 h before the first medium change, which was performed with special care to prevent the explants from detaching and facilitate effective cell outgrowth. At a cell confluence of approximately 90%, the explants were removed, and the cells were cultured in standard medium for further expansion until P4.

### Spheroid size and cell density optimization

For the preparation of spheroids, cells were detached with trypsin-EDTA, centrifuged, and resuspended in 200 µL of the appropriate medium for the respective experiment in an ultra-low attachment (ULA) plate (NunclonTM SpheraTM 96-Well, Nunclon Sphera-Treated, U-shaped-Bottom Microplate, ThermoFisher Scientific, USA). To determine the optimal cell density of spheroids, 0.5 × 10^4^, 1 × 10^4^, 2.5 × 10^4^, 5 × 10^4,^ and 10 × 10^4^ nasal or auricular CCs or CPCs (nas/aurCC/CPC) per construct were seeded in triplicates in commercially available chondrogenesis-inducing medium StemMACS™ ChondroDiff Medium (abbreviated as ChDif; Miltenyi Biotec, Bergisch Gladbach, Germany). Spheroids were then incubated at 37 °C and 5% CO_2_ for 21 days. Medium changes were performed every 48 h to 72 h.

### Viability and mitochondrial adenosine triphosphate (ATP)

To monitor cell viability, SYTOX™ Green Nucleic Acid Stain (from here onwards SYTOX, Invitrogen, Thermo Fisher Scientific Inc., MA, USA) was added to the spheroid cultures (dilution 1:10,000) for identification of dead cells. Viability was monitored on days 3, 7, 14, and 21. Furthermore, in experiments with CDM, on culture day 21, spheroids were stained with 5 µM BioTracker ATP-Red Live Cell Dye (Sigma-Aldrich) for fluorescent labeling of mitochondrial ATP and evaluation of metabolic activity and overall cell health. Images were acquired using an inverted fluorescence and bright field microscope Axio Observer (Zeiss, Germany) equipped with an Axiocam 503 (Zeiss, Germany) digital camera.

### Micromass cell culture

For micromass (MM) cultures, a droplet of 15 µL suspension containing 2.5 × 10^4^ or 30 × 10^4^ cells was pipetted into the center of each well of a 24-well plate, as previously described^23^. After 5 h of incubation at 37 °C, the MMs were carefully covered with ChDif or chondrogenic differentiation medium supplemented with 10 ng/mL TGF-β1 (Table 1) while avoiding the detachment of the formed cell aggregate and incubated for 21 days at 37 °C and 5% CO_2_. Regular medium changes every 48 h to 72 h were performed. The experiment was repeated with three donors in independent experiments.

**Table 1:** Composition of the tested chondrogenic media.

CDM cytokine composition	Vendor	Concentration
TGF-β1	PeproTech (NJ, USA)	10 ng/mL
TGF-β3	Proteintech (IL, USA)	10 ng/mL
CTGF	PeproTech	50 ng/ml
IGF-1	PeproTech	76 ng/ml
IGF-1 + CTGF	PeproTech	76 ng/ml + 50 ng/ml
StemMACS™ ChondroDiff	Miltenyi Biotec	--

### Comparison of chondrogenic differentiation media (CDM) with different cytokines

To test the influence of various cytokines on chondrogenic re-differentiation of nasal and auricular CCs and CPCs in a 3D culture, spheroids containing 2.5 × 10^4^ cells were cultured in five types of CDM containing four different cytokines as a mono-supplement or in combination, as listed in Table 1. All CDM combinations were based on chondrogenic medium consisting of DMEM with 4,5 g/l D-Glc, 1% L-Glutamine, 1% Pyruvate (Thermo Fisher Scientific Inc., Waltham, MA, USA), 5% FBS, 1% L-ascorbic acid (6.4 mg/ml), 0,1% dexamethasone (40 µg/ml) and 0,1% L-proline (40 mg/ml). Cytokine concentrations (Table 1) were estimated according to previous reports in the literature^16,18,19,32^. ChDif medium was used as a reference. Three healthy donors of nasal and auricular CCs and CPCs were tested in independent experiments.

### ATP measurements

To evaluate the influence of IGF-1 on the ATP levels in auricular and nasal CCs and CPCs, the cells were plated in 96-well plates in ChDif medium or CDM with IGF-1 (Table 1) at an equal density of 2000 cells per well and stained with 5 µM BioTracker ATP-Red Live Cell Dye. Fluorescent images were acquired at 4, 24, 48, 72, 144, 196, and 240 h. Furthermore, all cell types were seeded in ChDif medium or CDM with IGF-1 on a separate 96-well plate and ATP concentration at the initial time point of 4 h (before the onset of cell proliferation) was evaluated using the ATP Cell Viability Luciferase Assay (Merk, Germany), according to manufacturer’s instructions. Luminescence was measured using the Infinite 200 PRO multimode plate reader (Tecan, Austria).

### Histology and immunohistochemistry

Spheroids from two separate experiments with different donors were used for immunohistochemistry (IHC) staining. Spheroids were collected after 21 days of culture and fixed in 4% neutral buffered formalin. The MM were harvested by trypsinization and careful scraping with a metal spatula and washed immediately to preserve their integrity before fixing in 4% formalin. The fixed spheroids and MM were then embedded in agarose. After hardening, agarose-embedded cell aggregates were embedded in paraffin and sectioned at 5–7 μm. The slides were incubated with endogenous peroxidase-blocking solution, washed, and blocked with 10% normal sheep serum for 30 min. The serum was then removed by washing and the slides were incubated at 4 °C overnight with primary antibodies against collagen type II 1:100 (AB_528164, Developmental Studies Hybridoma Bank, USA), collagen I 1:200 (ab34710, abcam, UK), elastin 1:500 (ab23747, abcam, UK) or aggrecan 1:100 (AB1031, Merck Millipore, Darmstadt, Germany). The slides were then washed in PBS 0.1% Tween 20 before adding the corresponding biotinylated secondary antibody (GE Healthcare) for 45 min. The sections were then washed again and biotinylated horseradish peroxidase complex (GE Healthcare) was applied before the addition of 3,3′-Diaminobenzidine (DAB) solution (Scytek, Germany) and counterstaining the nuclei with Harris Hematoxylin Solution. Elastic staining was performed using the Sigma-Aldrich Staining Kit (HT25, Sigma-Aldrich, Germany) according to the manufacturer’s instructions.

Glycosaminoglycans (GAGs) were visualized by Alcian Blue staining. Briefly, paraffin sections were immersed in 1% Alcian Blue solution in 3% acetic acid (aa, pH 2.5) for 30 min at RT. The samples were then transferred to 3% acetic acid for 1 min and washed in distilled water for 2 min before counterstaining with 0.1% nuclear fast red dye (Sigma-Aldrich).

### TUNEL staining

**Fig. 1:**
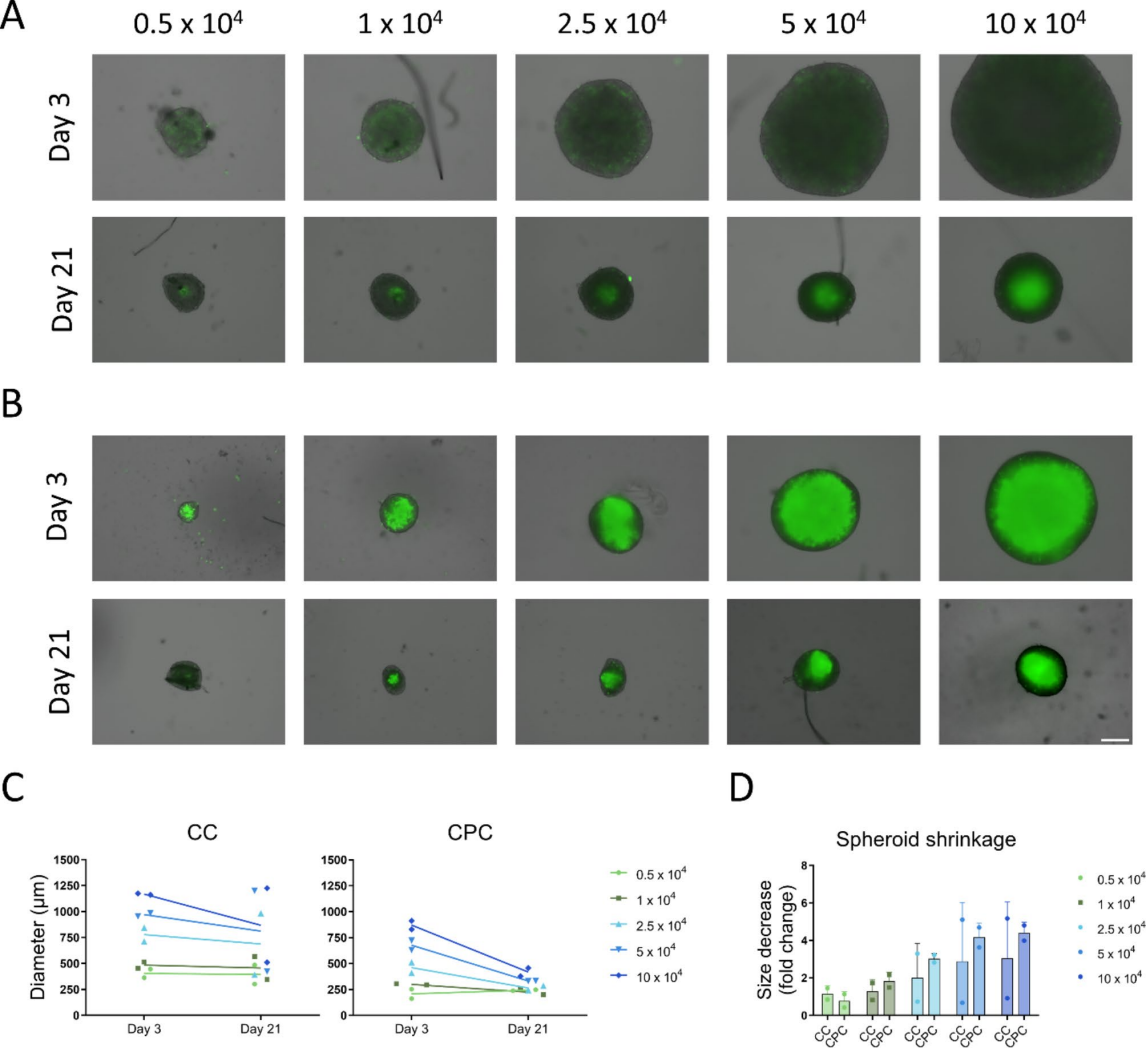
Optimization of spheroid density. (**A**). Micrographs depicting nasal chondrocyte (CC) and (**B**). chondroprogenitor (CPC) spheroids prepared with different cell densities after 3 and 21 days of culture in the ChDif medium. An overlay of brightfield images and SYTOX™ Green fluorescent staining of non-viable cells are shown. Scale bar 200 μm. (**C**). Spheroid diameters at culture days 3 and 21. (**D**). Fold decrease in spheroid size (shrinkage) calculated by obtaining the ratio of spheroid areas from culture day 3 and day 21. Data mean ± SD of 2 donors.

**Fig. 2:**
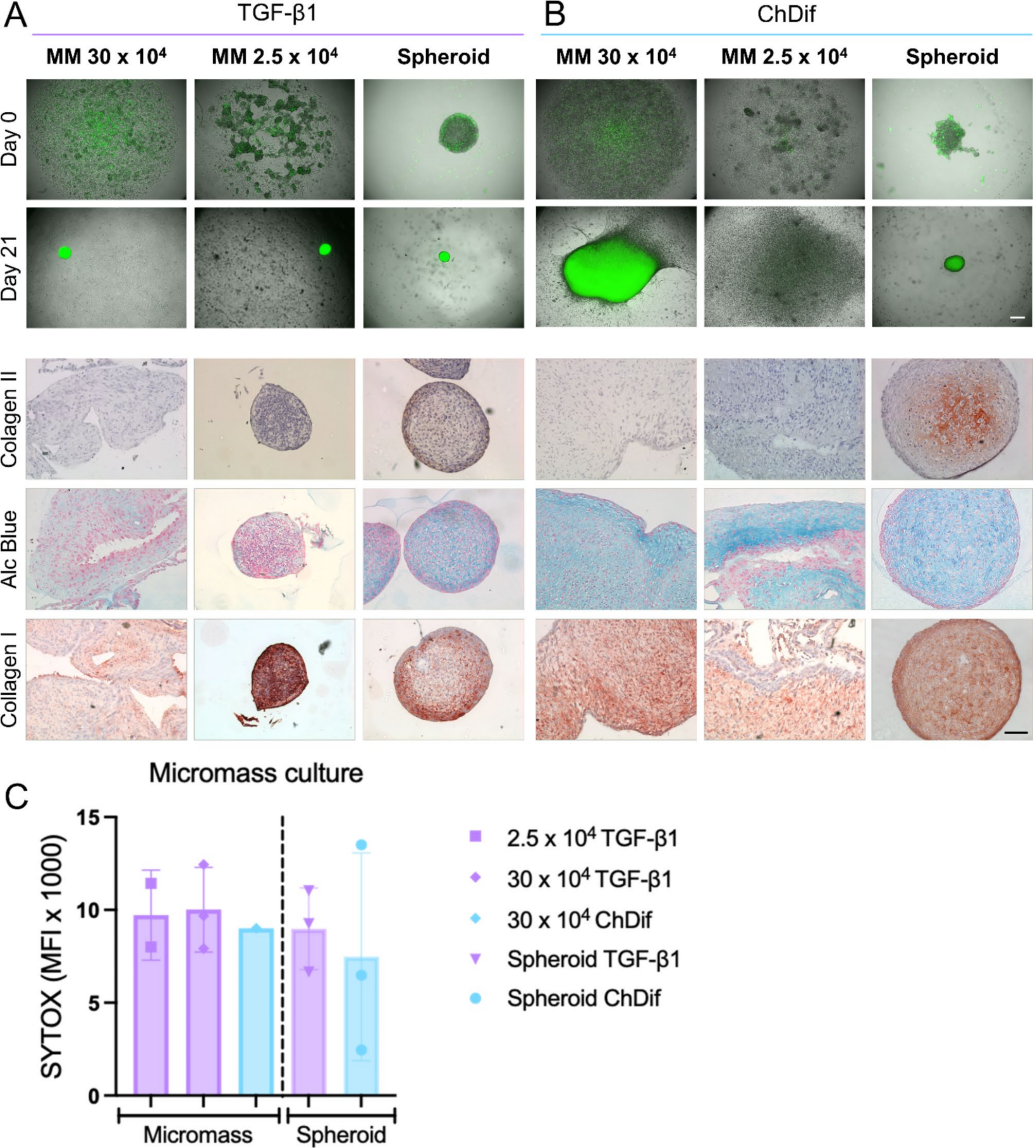
Comparison of micromass and spheroid cultures. (**A**). Non-viable cell staining (SYTOX™ Green) of micromass (MM) aggregates of nasal chondrocytes (CC) cultured in standard medium supplemented with TGF-β1 or (**B**). in commercially available chondrogenic differentiation medium (StemMACS™; ChDif). Spheroids prepared with 2.5 × 10^4^ cells were cultured for reference in both culture conditions (indicated as spheroid). All micrographs were acquired on the day of aggregate formation and culture day 21. Scale bar 500 μm. After 21 days of culture, all cellular aggregates were fixed and stained collagen type I and II or with Alcian Blue to visualize glycosaminoglycans (GAGs). Scale bar 100 μm. (**C**). Mean fluorescence intensity of all formed aggregates. MM aggregates with 30 × 10^4^ were only formed in one donor after 14 days, whereas no MM aggregates were formed in the ChDif medium at a lower cell density. Differences between conditions, as measured by two-way ANOVA, are not significant. Data – mean ± SD of 3 donors.

**Fig. 3:**
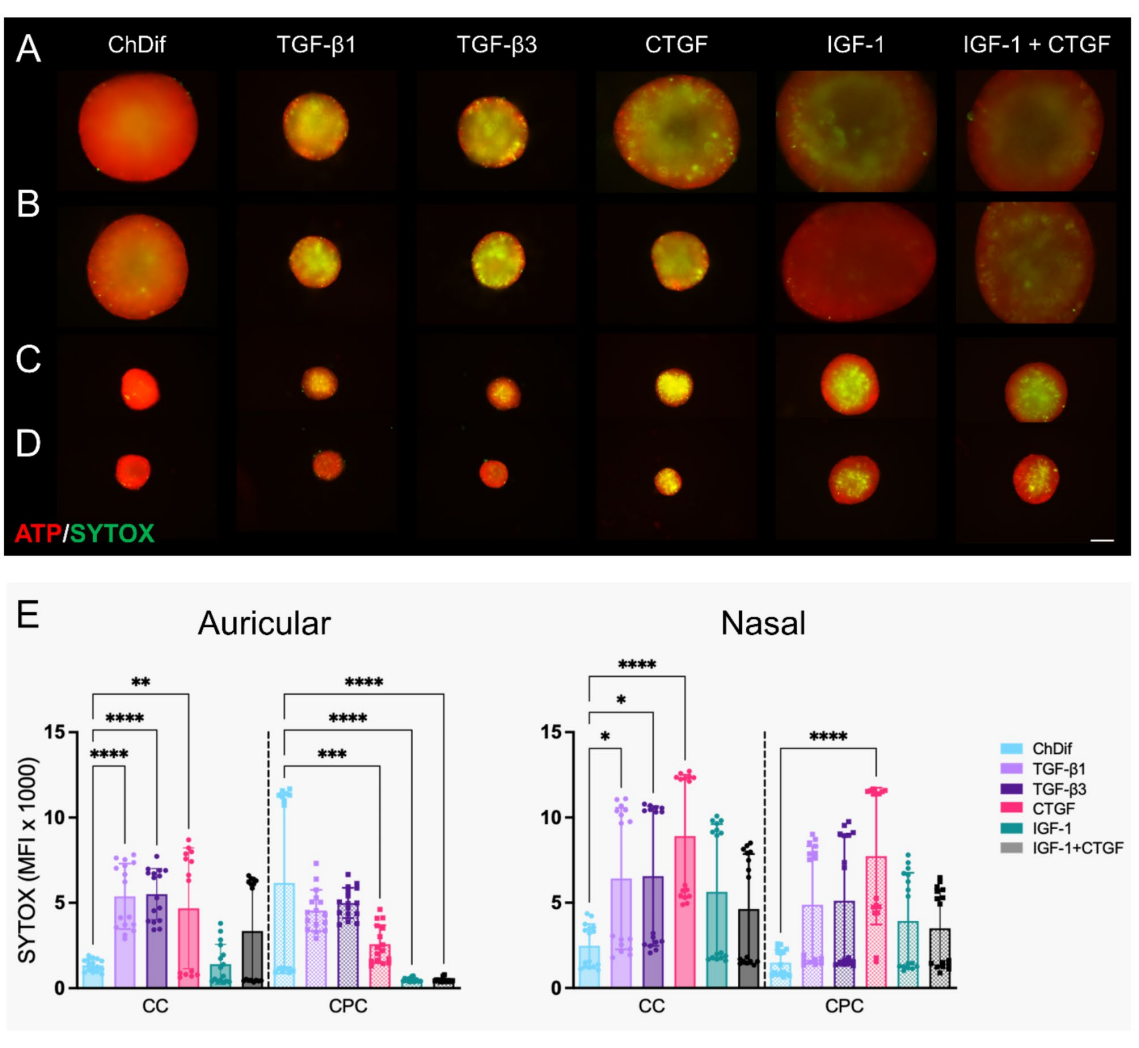
The influence of cytokine on HNC spheroid viability. Micrographs depict (**A**). auricular chondrocyte (CC), (**B**). auricular chondroprogenotor (CPC), (**C**). nasal CC, and (**D**). nasal CPC spheroids on culture day 21 in different CDM stained with BioTracker ATP-Red Live Cell Dye and SYTOX™ Green for non-viable cells. Scale bar 200 μm. (**E**). Mean fluorescence intensity (MFI) of SYTOX™ Green staining from auricular and nasal spheroids on culture day 21 in different CDM. All spheroids were formed at an established starting number of 2.5 × 10^4^ cells. Significance against ChDif, ****p < 0.0001, ***p < 0.001, **p < 0.01, *p < 0.05, One-way ANOVA. Mean ± SD of 8 spheroids from 2 different donors per cell type.

**Fig. 4:**
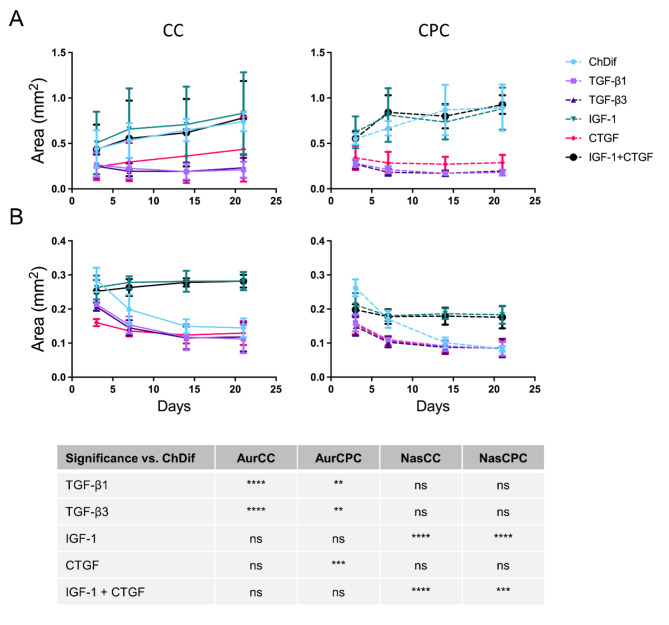
The influence of cytokines on HNC spheroid growth and size. Changes in the size of (**A**) auricular and (**B**) nasal chondrocyte (CC, solid line) and chondroprogenitor (CPC, dashed line) spheroids. Results from culture days 3, 7, 14, and 21 in six different conditions (Table 1) are shown. All spheroids were formed at an established starting number of 2.5 × 10^4^ cells. Mean ± SD of 8 spheroids from 2 different donors per cell type. The table shows the comparison of different conditions to the standard chondrogenic differentiation medium (ChDif) from graphs (**A**) and (**B**) ****p < 0.0001, ***p < 0.001, **p < 0.01, *p < 0.05, ns – non-significant, two-way ANOVA. Only results for day 21 are shown.

To confirm the location of non-viable cells in spheroid sections, fragmented DNA was labeled using the terminal deoxynucleotidyl transferase-mediated dUTP nick-end labeling (TUNEL) assay. TUNEL procedure was performed on fixed spheroid samples using the TUNEL Assay Fluorescence Kit (#25879, 488 nm, Cell Signaling Technology, USA) according to the manufacturer’s instructions. Total cell nuclei were counterstained using a DAPI staining solution (ab228549, Abcam) (Figs. 1, 2, 3 and 4).

### Image analysis

To analyze the viability, mitochondrial ATP staining, and spheroid size, we analyzed fluorescence and brightfield images acquired from two different focal (z) planes and at least 4 spheroids obtained from two different donors. The region of interest (ROI) was drawn around each spheroid and the Mean Fluorescence Intensity (MFI) and area of the ROI were measured using ImageJ software, version 1.54f (National Institutes of Health, USA). For every image, the background MFI of a region outside the spheroid was measured and subtracted from the spheroid MFI using Microsoft Excel software. Spheroid shrinkage (fold change in spheroid size) was calculated by obtaining a ratio between the spheroid areas measured on culture day 3 and culture day 21.

To measure corrected total cell fluorescence (CTCF) for the ATP measurement in monolayer culture, a threshold was set in the ATP channel (Triangle method) to exclude the background, the uneven background was removed manually, and the area, mean gray value, and integrated density were measured using ImageJ software. Per each image, the mean gray value of three background regions was measured and averaged to obtain the mean fluorescence of background readings. CTCF value was obtained using the formula CTCF = Integrated Density – (Area of selected cell X Mean fluorescence of background readings)^33^.

### Statistics

**Fig. 5:**
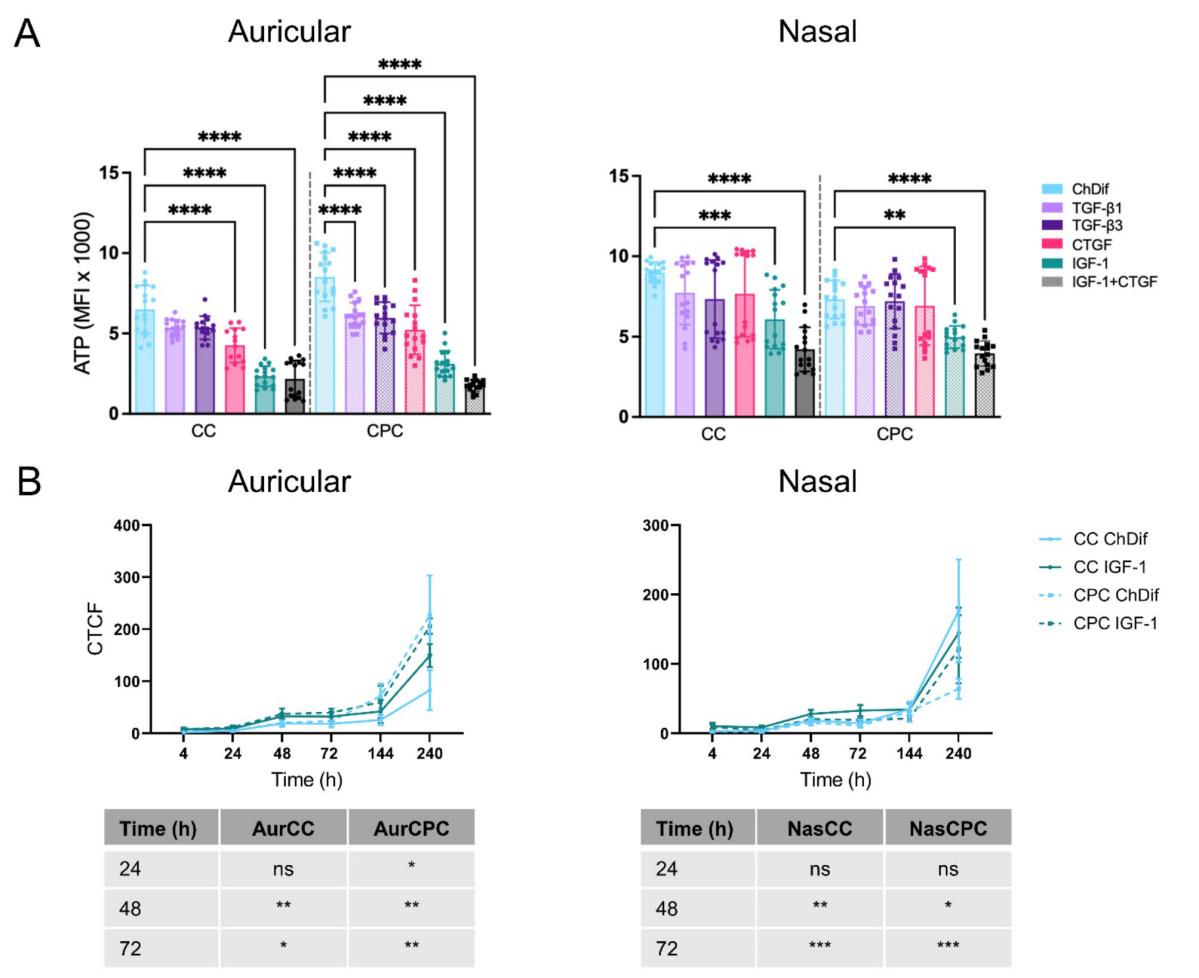
Mitochondrial ATP staining. (**A**). Mean fluorescence intensity (MFI) of auricular and nasal spheroids stained with BioTracker ATP-Red Live Cell Dye after 21 days of culture in different chondrogenic media (CDM), as depicted in Fig. 3A-D. Mean ± SD of 8 spheroids from 2 different donors per cell type. Significance against ChDif, One-way ANOVA, ****p < 0.0001, ***p < 0.001, **p < 0.01. (**B**). Corrected total cell fluorescence (CTCF) of ATP in auricular and nasal CCs and CPCs cultured 2D in ChDif medium or CDM with IGF-1 for 240 h, Mean ± SD of 3–11 micrographs. Significance values by mixed effect model followed by Tukey’s post hoc analysis displayed as follows - ***p < 0.001, **p < 0.01, *p < 0.05, ns – non-significant. Comparisons at earlier and later time points are not significant and are not shown on the tables.

The statistical significance of all samples was analyzed using GraphPad Prism version 9.5.1 for Windows (GraphPad Software, USA) by one- or two-way analysis of variance (ANOVA), as indicated in the figure caption. In the ATP study with 2D cultures (Fig. 5), the number of quantifiable images acquired from different wells varied between samples and at different time points. As ANOVA cannot handle missing values, the comparison of repeated measures in such cases was performed by fitting a mixed model with the Geisser-Greenhouse correction as implemented in GraphPad Prism 8.0^34^. This mixed model uses a compound symmetry covariance matrix and is fit using Restricted Maximum Likelihood (REML). In the absence of missing values, this method gives the same P values and multiple comparison tests as repeated measures ANOVA. In the presence of missing values (missing completely at random), the results can be interpreted like repeated measures ANOVA.

## Results

### Optimization of spheroid size and cell density

An important consideration for spheroid culture is the choice of optimal cell numbers. The goal is to use the minimum number of cells necessary to produce stable aggregates of a sufficient size for easy handling and analysis, without requiring unnecessary 2D cell expansion. To estimate the minimal cell density for producing viable aggregates with stable shape and sufficient size, we prepared nasal CC and CPC spheroids with cell numbers ranging from 0.5 to 10 × 10^4^ per single construct and cultured them in the ChDif medium. Nasal spheroids tend to be relatively small compared to auricular aggregates^35^, which determined our choice for nasal CC and CPC in this experiment.

Spheroids formed with cell numbers as low as 0.5 × 10^4^ and increased proportionally in size with higher cell densities (Fig. 1A and B, Figure S1A). In most spheroids, a reduction of the initial size (shrinking) was observable already by culture day seven and progressed until day 21. Typically, size reduction was more pronounced in larger spheroids, and was observable in CPC spheroids from both donors, as well as CC constructs from donor 1 (Fig. 1A and B, Figure S1A). The area of CPC spheroids was reduced by 3-fold for medium-sized aggregates prepared from 2.5 × 10^4^ cells and by more than 4-fold for larger spheroids containing 5 × 10^4^ and 10 × 10^4^ cells (Fig. 1C and D). In contrast, small CPC spheroids containing 1 × 10^4^ cells shrunk by < 2-fold, while the smallest CPC spheroids prepared from 0.5 × 10^4^ cells remained constant or even slightly increased in size (Fig. 1C and D). Due to the strong shrinkage of larger spheroids, the differences between different spheroid diameters were reduced by culture day 21, constituting 243.5 ± 7.2 μm for the smallest, 264.4 ± 29.3 μm for medium (2.5 × 10^4^) and 417.5 ± 55.2 μm for the largest CPC spheroids (Fig. 1C).

Unlike CPCs, CC spheroids showed contrasting behavior between the two donors. Although both donors formed similar-sized spheroids per given cell density (Fig. 1A and B, Figure S1A) aggregates from donor 1 shrank similarly to their CPC counterparts (Fig. 1), while those from donor 2 increased slightly in size irrespective of the cell density (Figure S1A, Fig. 1C and D). The shrinkage pattern in donor 1 CC spheroids resembled that of the CPC aggregates but with an even more pronounced reduction of the initial size than observed with CPCs. Namely, the size of larger spheroids (5 and 10 × 10^4^) was reduced by more than 5-fold, while a 3-fold reduction from the initial size was seen in smaller spheroids with 2.5 × 10^4^ cells, and less than 2-fold reduction was observed in the smallest spheroid with 1 and 0.5 × 10^4^ cells (Fig. 1D).

As expected, auricular CCs and CPCs formed larger spheroids than nasal cells. Spheroids from both types of auricular cells exhibited growth in culture with no tendency to shrink, forming substantially large constructs by day 21 even with a cell density as low as 1 × 10^4^ (Figure S1B).

In summary, consistent shrinkage-growth patterns were observed among medium-sized (2.5 × 10^4^) and large nasal spheroids (5 and 10 × 10^4^), while lower cell numbers (0.5 and 1 × 10^4^) formed small spheroids with very little size change over 21 days in culture, probably indicative of low cell-cell interaction and matrix remodeling within the aggregates. Furthermore, aggregates formed with 2.5 × 10^4^ cells were sufficiently large and exhibited comparable viability to their larger counterparts. Given these factors, 2.5 × 10^4^ cells per spheroid were assumed as an optimal minimal cell number for nasal spheroids. For consistency, auricular spheroids were also prepared with a similar cell density throughout the study.

Finally, we also assessed whether spheroids at this density could fuse into larger constructs when co-cultured. We repeatedly observed a complete fusion of constructs with 2.5 × 10^4^ cells, which typically took up to 12 days to complete (Figure S2).

### Comparison of MM and spheroid culture

To investigate if alternative scaffold-free culture methods could effectively generate cell aggregates with the established minimal cell density of 2.5 × 10^4^, we conducted micromass (MM) cultures using nasal CCs with comparable cell numbers. Alternatively, we prepared MM cultures with a high cell density of 30 × 10^4^ cells, approximating the average numbers reported in previous studies for MM culture^21–23^. Both types of scaffold-free cultures were cultivated in ChDif or CDM supplemented with TGF-β1 (Table 1).

In contrast to spheroids, the aggregation of cells in MM cultures was often delayed or even ineffective. The aggregates were only visible on the third day in MM culture with TGF-β1, while spherical self-assembly was visible already at the start of the culture on low-attachment plates (Fig. 2A, Figure S3A). The viability of cells in both MM and spheroid cultures in TGF-β1 medium was visibly compromised, as indicated by the increased intensity in the SYTOX nuclear stain (Fig. 2C). Although the cell density of the MM culture did not seem to affect the viability of cell aggregates in the TGF-β1 medium, it did affect the shape of the aggregate, which occasionally deviated from a spherical structure and acquired an elongated form (Figure S3).

The behavior of both cell aggregate types was different in ChDif as compared to the culture with TGF-β1 supplementation. Namely, a complete lack of cell aggregation was observed in MM cultured in ChDif medium at a density of 2.5 × 10^4^ and only delayed cell aggregation with a formation of non-spherical, spread-out cellular structure was observed at a higher density of 30 × 10^4^ (Fig. 2B). In some donors, no MM aggregate formation was observed even at higher cell density in ChDif (Figure S3B). In contrast, spheroids from all donors formed effectively in the ChDif medium on the ULA surfaces. Furthermore, unlike spheroids, an outgrowth of cells attaching to the plastic was usually observed in large MM structures, which disrupted the sphericity of the aggregate structure (Fig. 2B).

The IHC analysis revealed no collagen II staining in MM cultured in TGF-β1 medium at any cell density, whereas moderate positive staining for collagen II was detected at the edges of spheroids (Fig. 2A). A limited presence of GAGs was only observed in spheroids and, to a lesser extent in MM with 30 × 10^4^ cells in TGF-β1 CDM, as represented by an inhomogeneous and weak Alcian Blue stain.

Consistently with the lack of effective cell aggregation, MM cultures of any cell density also did not show positive collagen II staining after 21 days in the ChDif medium. In contrast, the collagen II-positive area was observed centrally in spheroids cultured in ChDif on ULA plates (Fig. 2B). Alcian Blue staining was also more pronounced in ChDif cultures, particularly in spheroids and partially formed MM aggregates with 30 × 10^4^ cells. Collagen I staining was positive in all 3D aggregates, regardless of the culture condition, but was especially high in low-density MM with 2.5 × 10^4^ cells (Fig. 2A).

Overall, the formation of spherical cell aggregates was more efficient on ULA plates as compared to the MM method, which could not facilitate effective cell aggregation at a lower cell density. Additionally, chondrogenic differentiation was observed in nasal spheroids cultured in ChDif medium, but not in nasal MM, as indicated by the production of GAGs and collagen II.

### The influence of cytokines on HNC spheroid size, growth dynamics, and viability

The composition of the culture medium is crucial for the successful in vitro maintenance of cartilage spheroids. To evaluate the maturation and growth of HNC spheroids under varying conditions, we supplemented CDM for spheroid culture with different cytokines that are commonly used in chondrogenic cultures (Table 1). ChDif medium was used as a reference throughout the study, as a standard commercially available medium for chondrogenic differentiation.

CDM composition impacted spheroid characteristics, such as size, and viability. Differences in the response to cytokines were observed between auricular and nasal cells, and to some degree, between CCs and CPCs from the same tissues. In all conditions, auricular cells generated overall larger spheroid constructs as compared to the nasal counterparts (Figs. 3A–D and 4). Non-viable cell staining of spheroids from both tissues varied among donors. In particular, auricular donor 1 displayed pronouncedly high SYTOX staining selectively for CC spheroids (Figure S4A), while nasal donor 2 showed poor viability of spheroids prepared from both CC and CPCs (Figure S4B). Nevertheless, the influence of medium composition on viability was discernable with culture time, whereby the donors with poor viability pronouncedly reduced the SYTOX staining in favorable conditions (e.g. ChDif, or IGF-1-containing media), reflecting the tendencies observed in donors with good viability (Figure S4). As a general trend, CPCs from both tissues displayed lower staining for non-viable cells. While similarities were observed between the response of nasal and auricular spheroids concerning size and growth dynamics, the extent of visible cytokine influence differed among the two tissues. The details on the characteristics of different HNC spheroids are described below separately per each tested condition.

All spheroids showed good viability in the ChDif medium relative to other CDM conditions (Fig. 3). Lower staining for non-viable cells was observed in auricular spheroids, particularly in the early culture period (Figure S 4A). The tendency of decrease in SYTOX signal with culture time was observed in spheroids from both tissues, except for aurCPC donor 1, which showed a sudden drastic increase in the SYTOX staining at days 14 and 21 (Figure S4A, Fig. 3E). Irrespective of the viability, auricular spheroids showed a gradual increase in size (Figs. 3A and B and 4A). By day 21 in the ChDif medium, the diameter constituted on average 969 ± 65.7 μm for aurCC spheroids and 1057 ± 143.3 μm for aurCPC spheroids.

In contrast, nasal spheroids, which were substantially smaller than their auricular counterparts (Fig. 3C and D), shrank further with culture time (Fig. 4B). Here, CPC spheroids were smaller than CCs and the difference in size became even more apparent at later culture time points, with CPC aggregates shrinking more in comparison to CC spheroids (Fig. 4B). By day 21, nasCC spheroids measured 427 ± 38.9 μm and nasCPC constructs reached the average diameter of 325 ± 15.7 μm. Importantly, in contrast to the experiments with the cell density (Figure S 1 A), all nasCC spheroids cultured in ChDif in this experiment exhibited shrinking behavior without an exception.

The two growth factors of the TGF-β family had very comparable effects on the size and viability of different spheroids. For both TGF-β1 and TGF-β3, auricular and nasal spheroids alike showed relatively high SYTOX staining (Fig. 3E), which reduced only slightly with culture time (Figure S 4) and even increased in the case of aurCPCs (Figure S 4A). Auricular spheroids cultured in a media with TGF-β1/3 were distinctively small (Fig. 3A and B) and showed no change in size during the culture period, irrespective of changes in viability (Fig. 4A). Nasal CCs and CPCs showed some shrinkage in TGF-β media but the size decrease was more moderate as compared to nasal spheroids cultured in ChDif (Fig. 4B).

CTGF is a downstream effector of TGF-β^36^ and stimulates the expression of IGF-1 in chondrocytes^37^. Furthermore, it is known to bind and synergize with both IGF-1 and TGF-β in different physiological and pathologic conditions^37^. In auricular samples, CTGF produced spheroids with intermediate characteristics in terms of viability and size as compared to those grown in TGF-β1/3 and all other CDM. Namely, CTGF spheroids were slightly larger (Figs. 3A and B and 4A) and showed somewhat lower SYTOX staining (Fig. 3E) than TGF-β1/3 aggregates but were smaller and less viable as compared to other CDM conditions. CTGF-grown nasal spheroids overall resembled those cultured with TGF-β1/3 in terms of size and shrinkage patterns (Figs. 3C and D and 4B) but showed higher SYTOX signal compared to TGF-β1/3 counterparts (Fig. 3E).

IGF-1 is an anabolic cytokine, which is produced by chondrocytes and, similarly to the CTGF plays an important role in chondrocyte proliferation, GAG synthesis, and maintenance of phenotype^37,38^. In terms of spheroid size, supplementation of medium with IGF-1 yielded very similar spheroids to those cultured in IGF-1 and CTGF combination.

A strikingly pronounced effect of IGF-1 was observed on the size of auricular spheroids cultured with IGF-1 alone or in combination with CTGF (Fig. 3A and B). As with ChDif, the distinctively large size of auricular spheroids in IGF-1-containing CDM was apparent already at culture day 3 and progressively increased with culture time (Fig. 4A). Non-viable cell staining was also comparable to that of auricular spheroids in ChDif typically displaying low MFI values for SYTOX (Fig. 3E).

Nasal spheroids cultured with IGF-1-containing media were not as large as auricular aggregates but were nevertheless the largest among spheroids in all conditions compared (Fig. 3C and D). Furthermore, with IGF-1 only negligible shrinkage was observed after culture day 3 in nasal CPC aggregates, while no shrinkage and even slight growth was observed in nasal CC spheroids, which were on average larger than their CPC counterparts (Fig. 4B). Stable viability of both nasal CC and CPC spheroids was observed in IGF-1-containing media (Fig. 3E), with a low SYTOX signal in donor 1 and a reducing SYTOX signal in donor 2 (Figure S4B).

### The influence of cytokines on mitochondrial ATP

ATP is the primary source of cellular energy. It is commonly used as an indirect indicator of metabolic activity, overall cell health, and viability. To confirm the presence of viable, metabolically active cells within HNC spheroids at culture day 21, the aggregates were labeled with a mitochondrial ATP-binding fluorescent dye. Positive staining of ATP was observed in all spheroids, confirming the presence of viable cells within aggregates (Fig. 3A-D). However, the intensity of the mitochondrial ATP signal across various conditions was not always reflective of SYTOX non-viable cell staining. Namely, ChDif cultures had the greatest mitochondrial ATP signal in both auricular and nasal (Fig. 5A) spheroids, including spheroids with high SYTOX staining (e.g. AurCPC, donor 1). In contrast, spheroids in IGF-1-containing media showed the weakest staining signal of mitochondrial ATP (Fig. 5A**)**.

Similarly to insulin, IGF-1 is implicated in glucose transport and oxidation in vitro^38,39^. We, therefore, hypothesized that IGF-1 could have affected the level of mitochondrial ATP through its influence on metabolic activity in the individual cells, with subsequent changes in the ATP staining signal.

To test the influence of ATP on individual cells, we cultured all cell types in 2D in either ChDif medium or CDM supplemented with IGF-1 and measured the ATP signal using a luminescence-based assay. Furthermore, the 2D cultures were also stained using the ATP-Red Live Cell Dye, to ensure that our staining of mitochondrial ATP was reflective of the intracellular ATP levels. ATP measurements using a luminescence-based assay were performed immediately after cell attachment at an early time point of 4 h to exclude the increase of ATP due to cell proliferation. In contrast to the spheroid cultures, the measurements of the luminescence-based assay showed a significant increase in ATP levels in all IGF-1 samples, which was particularly pronounced in the aurCCs and even more so in aurCPCs (Figure S5).

To evaluate the dynamic changes in the ATP levels in IGF-1 CDM or ChDif monolayer cultures, we monitored the cells with ATP-binding fluorescent dye until maximal cell confluence. The ATP levels showed a steady increase in all conditions and cell types (Fig. 5B, Figure S6). Consistent with the luminescence measurements, the strongest fluorescence was detected in aurCPCs followed by aurCCs (Fig. 5B). Significantly higher ATP staining was observed in both auricular and nasal CCs and CPCs cultured in IGF-1 CDM as compared to ChDif after 48 h and 72 h (Fig. 5B). As the cells reached confluence at later time points, the overall intensity of fluorescence staining in both conditions began approximating each other. Nevertheless, the differences between the cells cultured in ChDif and IGF-1 CDM were discernable. In particular, ChDif cultures reached a higher density of cells and were confluent by 144 h and over confluent by 240 h (Figure S6), whereas CCs and CPCs in IGF-1 CDM proliferated less, reaching confluence by 240 h, and showed a more granular, defined ATP staining pattern than the cells in ChDif.

Overall, while low ATP staining was observed in spheroids grown in IGF-1-containing CDM, monolayer cultures showed that IGF-1 increases ATP staining in individual cells.

### Assessment of cell viability by TUNEL assay

TUNEL assay has been widely used to detect cell-death-associated DNA fragmentation and can be combined with IHC in formalin-fixed paraffin-embedded (FFPE) tissues^21^. Because the conventional imaging method has limitations in evaluating viability, such as the inability to visualize individual cells, and due to the impact of culture medium composition on ATP viability staining as described above, we conducted the TUNEL assay on FFPE auricular spheroids to validate the viability monitoring of live samples in our study ( as shown Fig. 3). Overall, the distribution of TUNEL-labeled nuclei in the spheroid sections was relatively similar to the SYTOX labeling pattern. Similarly, the ATP label corresponded well with the distribution of total cell nuclei within the spheroid (Figure S7).

### The influence of cytokines on spheroid morphology and ECM

To assess the quality of chondrogenic cultures, we performed IHC staining of spheroids grown for 21 days in CDM with various cytokine supplementations (Table 1). The spheroids were stained for key cartilage ECM constituents, such as collagen II, aggrecan, and elastin. Furthermore, staining for collagen I was performed to evaluate the tendency for fibrocartilage formation. Positive staining for collagen I was observed in all spheroids regardless of CDM conditions of cell type (Figure S8).

**Fig. 6:**
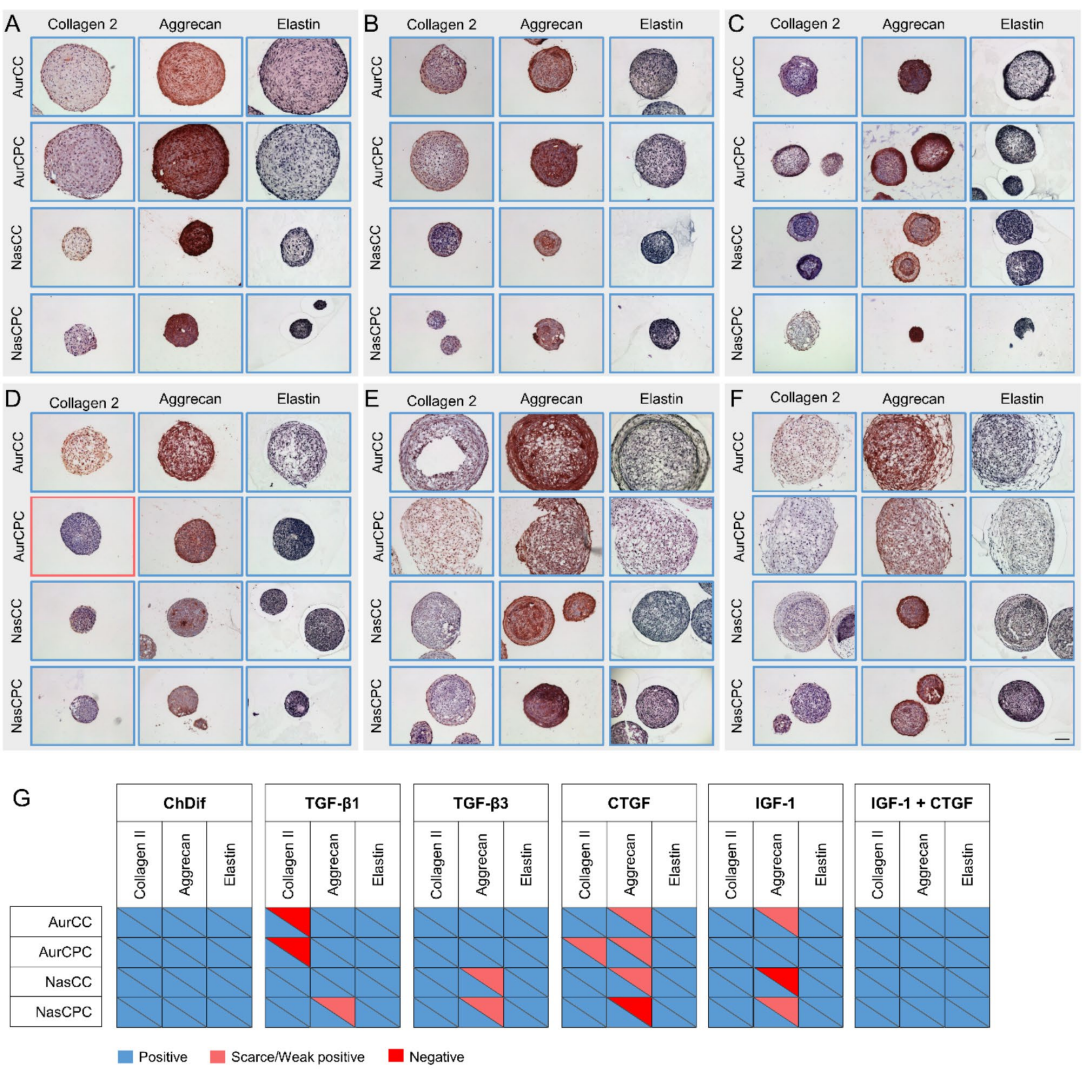
Spheroid structure and cartilage ECM components. Micrographs depict the results of immunohistochemistry (IHC) for collagen type II, aggrecan, and elastic stain in auricular and nasal chondrocyte (CC) and chondroprogenitor (CPC) spheroids cultured in (**A**). ChDif or chondrogenic differentiation medium (CDM) containing (**B**). TGF-β1, (**C**). TGF-β3, (**D**). CTGF, (**E**). IGF-1, or F. CTGF + IGF-1. The color of the micrograph border corresponds to the degree of staining, whereby positive staining is shown as blue, scarce/weak staining is shown as light red, and negative staining as dark red. All stainings in this experiment were positive. Scale bar 100 μm. Results from one of two independent experiments are shown. G. Summary of IHC results from two experiments (second in Figure S9). Each cell is divided into two parts representing the results from two donors. AurCC/CPC - auricular CC/CPC, nasCC/CPC - nasal CC/CPC, ChDif - StemMACS™ ChondroDiff Medium.

ChDif produced spheroids of moderate cellularity and density of ECM arrangement (Fig. 6A, Figure S9A). All cartilage ECM components tested were positive in all spheroids cultured in ChDif, showing a good chondrogenic capacity of the medium (Fig. 6G). Little to no structural patterns or characteristic distribution of ECM was observed.

TGF-β1 and TGF-β3 produced spheroids with a compact ECM structure, densely populated with cells, and often showed a cellular and ECM arrangement pattern. Particularly, in spheroids cultured with TGF-β1/3, a dense cell layer with a parallel arrangement was often observed surrounding an intermediate layer of cells with a more perpendicular, less orderly pattern and a loose necrotic core in the center (Fig. 6B and C, Figure S9B and C, Figure S10). This structure was most clearly pronounced in nasal CC spheroids (Figure S10).

Concerning chondrogenic differentiation, the presence of cartilage ECM was confirmed in all spheroids cultured in CDM with TGF-β3. In contrast, TGF-β1 induced the production of all tested ECM components in all spheroids, except for auricular aggregates from one of the two donors tested in our study, showing no detectable collagen type II in either aurCC or aurCPC in CDM with TGF-β1 (Fig. 6G, Figure S9B and C, Figure S10). All auricular spheroids grown in TGF-β1/3 showed positive aggrecan staining in both donors. In contrast, nasCPC spheroids from one donor displayed only scarce positive aggrecan staining in TGF-β1/3. Similarly scarce aggrecan staining was also seen in nasCC spheroids from the same donor (Fig. 6G, Figure S9B and C).

While CTGF produced relatively small spheroids with similar size and viability dynamics as TGF-β1/3, structurally the culture groups differed substantially. In contrast to TGF-β1/3, spheroids cultured in CTGF CDM had a looser structure, with randomly arranged cells surrounding an often amorphous and hypocellular core with scarce ECM content (Fig. 6D, Figure S9D, Figure S10).

NasCPC spheroids were an exception and were more compact than all other spheroid types cultured with CTGF, albeit lacking the characteristic outer layer of cells, as seen with TGF-β1/3 (Figure S 10). Remarkably, in one out of the two donors tested, CTGF led to no detectable aggrecan expression in nasCPCs and only very scarce aggrecan production in all other spheroids (Fig. 6D and G, Figure S 9D). Collagen II was detected in all spheroids grown in CTGF CDM, albeit only scarcely in aurCPC spheroids from one of the donors (Fig. 6D and G, Figure S9D). Elastin staining was positive and abundantly distributed throughout the spheroids (Fig. 6D and G, Figure S9D).

Spheroid structure and ECM content in spheroids cultured with IGF-1 were somewhat comparable to those of CTGF-grown aggregates. IGF-1 spheroids showed an even more hypocellular, loose ECM, with a pronounced sponge-like, aerated texture not only in the spheroid core but often throughout the aggregate structure (Fig. 6E, Figure S9E, Figure S10). In contrast to CTGF, and somewhat similar to TGF-β1/3, spheroids cultured with IGF-1 often displayed some discernable zonality particularly in CC aggregates, displaying a distinctive outer layer with a parallel arrangement of cells and ECM (Fig. 6E, Figure S9E, Figure S10). In contrast to TGF-β spheroids, however, here the outer layer was looser than the adjacent intermediate zone, which showed a perpendicular arrangement of cells and ECM and was more densely structured than the outer layer and the spheroid core. As with CTGF, nasCPCs had a relatively compact structure throughout the aggregate as compared to all other spheroid types in the same condition.

Further similarity with CTGF-grown spheroids was the predominantly low presence of aggrecan in one of the donors in the study. In these samples, IGF-1 culture resulted in scarce positive staining in aurCC and nasCPC, moderate staining in aurCPC, and no detectable staining in nasCC spheroids (Fig. 6G, Figure S9E). As with CTGF, collagen II and abundant elastin were found in all aggregates cultured with IGF-1 (Fig. 6E and G, Figure S9E).

A combination of the two cytokines produced large spheroids that showed morphological resemblance to both IGF-1 and CTGF-grown aggregates. Here, however, the abundant presence of aggrecan, elastin, as well as collagen I was detected throughout all spheroid types (Fig. 6F and G, Figure S9F, Figure S8). Similarly, strong collagen II staining was detected in auricular and nasal CC, nasCPC, and somewhat less so, in aurCPC spheroids (Fig. 6F, Figure S9F).

In summary, spheroids cultured in different CDMs showed distinctions in terms of architecture, as well as staining for cartilage ECM proteins. Albeit at varying degrees, the production of all cartilage ECM molecules was stimulated by ChDif, TGF-β3, and a combination of IGF-1 + CTGF in all constructs (Fig. 6G), with particularly superior immunoreactivity of all ECM components visualized by IHC in ChDif and IGF-1 + CTGF spheroids. Notably, however, the latter conditions also stimulated high collagen I production (Figure S8). In other conditions, some cartilage ECM components were absent or scarcely present in either one of the two donors tested in the study, such as collagen II in auricular spheroids cultured with TGF-β1, or aggrecan in nasCPC spheroids cultured with CTGF and nasCC spheroids grown in CDM with IGF-1.

## Discussion

Engineering cartilage that mimics native tissue structure, composition, and mechanical properties remains challenging. Top-down approaches, which employ single cells and biomaterials to obtain TE cartilage, often have considerable shortcomings associated with inhomogeneous cell distribution, the lack of physiological cell-cell and cell-matrix interactions, and the resulting deficiencies of mechanical properties and tissue architecture^40^. An alternative modular approach employs a bottom-up scaffold-free strategy taking advantage of the spontaneous self-assembly of cellular aggregates to create microtissues. These are then used as building blocks for TE tissue by fusion of microtissues into larger constructs or combining them with biomaterials. Research using this strategy has been recently emerging to improve the quality of articular cartilage TE^29,30,41^.

The scaffold-free approach has also been employed for the engineering of auricular cartilage, albeit mostly in the form of multilayered cell culture of human chondrocytes^4,42^ and perichondrocytes^43^, or cultivation of auricular chondrocytes and perichondrocytes under continuous medium flow in bioreactors^19,44^. Furthermore, micromass culture of auricular CCs^45^ or macroaggregate cultures of auricular and nasal CCs using cell culture inserts^46^ have been proposed to obtain bulk cartilage tissues. These approaches, however, require very high cell numbers^42,45,46^ or special equipment like bioreactors^19,44^ and often produce cartilage that lacks characteristics of native tissue ECM, such as elastin^19,45,46^.

More recently, microspheroids prepared from auricular perichondrial chondroprogenitors have been used to obtain auricular cartilage by fusion of spheroids in a rotating wall vessel bioreactor^28^. The resulting tissue exhibited some native-like features, such as elastin fibers and collagen II, further demonstrating the potential of a scaffold-free approach for HNC cartilage TE. Furthermore, the application of HNC spheroids can be extended to articular cartilage TE. For example, nasal septal CC spheroids have been suggested as a superior alternative to articular CC constructs for the repair of osteochondral defects in a rat model^47^.

In this study, we tested basic culture parameters, such as cell density and medium composition to establish minimal conditions for HNC spheroids that allow efficient redifferentiation of expanded CCs and CPCs. Cell density in particular could be a critical factor for the survival of chondrocytes.

Mature cartilage is typically a hypocellular tissue, with one or more chondrocytes surrounded by a pericellular matrix within a chondron. Thus, unlike MSCs, which undergo condensation during developmental processes and cartilage formation, CCs and CPCs from mature cartilage do not experience a high degree of cell-cell contact in vivo. Higher susceptibility of articular chondrocytes to apoptosis has been described in confluent monolayer cultures as opposed to low-density cultures, suggesting the importance of cell-cell contact in modulating apoptotic response^48^. The initial cell aggregation during spheroid formation in our study was indeed often characterized by high non-viable cell staining, albeit usually followed by stabilization of viability in culture. Naturally, for proper cell aggregation and subsequent microtissue formation, cell-cell contact cannot be avoided. However, the reduction of cell density may still influence apoptotic signaling by modulating the degree of cell-cell contacts. We, therefore, evaluated whether cell density in a 3D culture setting could potentially affect cell viability. However, no clear patterns showing the dependence of viability on cell density were observed in our spheroid cultures, with only seemingly incidental differences between individual spheroids irrespective of the initial cell numbers.

Nevertheless, given the tendency of CCs and CPCs to dedifferentiate with passaging, spheroids should ideally require minimal cell numbers and preliminary cell expansion in a conventional culture, while fulfilling several important criteria. Primarily, a robust assembly of aggregates with reproducible size and shape should capacitate straightforward procedures and easy handling in standard lab conditions, preferably, avoiding the need for sophisticated equipment for the assembly or maturation of the constructs. Following the assembly, ECM reorganization within the cellular aggregate typically manifests by a change in construct size as either growth or shrinkage, over a culture period. Furthermore, the common initial loss of viability during cell aggregation must be counterbalanced by proliferation, active metabolism, and deposition of proper ECM by the surviving cells. A small spheroid size that allows sufficient nutrient diffusion can be crucial in this respect besides biochemical factors, such as medium composition.

Using ULA plates, cell aggregation was observed with cell numbers as low as 0.5 × 10^4^. However, nasal spheroids prepared with cell density below 2.5 × 10^4^ were extremely small for ease of handling (e.g. embedding for IHC) and showed very little dynamic change in the aggregate size, possibly indicative of the lack of cell-cell signaling necessary for proper spheroid maturation and ECM reorganization. At a density of 2.5 × 10^4^ in a standard ChDif culture, the smallest spheroid type (i.e. nasCPC) was still sufficiently large for handling while not requiring high cell numbers for preparation and exhibiting growth dynamics similar to that of larger aggregates, suggesting that sufficient paracrine signaling and cell-cell contacts were present. Collectively, the experiments in our study, however, emphasize that besides cell density, parameters such as cell isolation method and anatomical origin, as well as cytokine composition and ECM structure (discussed below in more detail), are all crucial factors when considering the construct size.

The shrinking behavior was mostly seen in nasal spheroids and contrasted culture dynamics of auricular aggregates, which showed growth or remained stable in size depending on the culture conditions. An exception was observed for only one donor, which showed slight growth for nasCC but shrinkage for nasCPC (Figure S1A) when cultured in ChDif. However, for all other donors within our study, both nasCC and nasCPC aggregates showed a reduction in size in most culture conditions. A possible reason for the shrinking of nasal spheroids could be the high expression of matrix-degrading enzymes. In comparison to auricular chondrocytes, nasoseptal chondrocytes are known to express high levels of MMP1, MMP2, MMP3, MMP13, as well as ADAMTS5 and cathepsin B^35,49^. Furthermore, the shrinking was more apparent and consistent across donors in nasCPC spheroids as compared to nasCCs. Whether this difference is caused by higher expression of matrix-degrading enzymes in nasCPC vs. nasCC, or if there are other factors in play, such as higher condensation capacity of nasCPC, remains to be elucidated.

A direct comparison of MM and spheroid cultures revealed that cell aggregation on ULA surfaces is a superior method for obtaining cartilage microtissues, particularly when the preparation of aggregates using low cell numbers is desired. Using the MM approach, aggregates with 2.5 × 10^4^ cells completely failed to form in the ChDif medium, whereas aggregates with 30 × 10^4^ cells formed only for one donor in the same condition. While MM did assemble in a medium with TGF-β1 supplementation, the assembly was delayed compared to the spheroids, producing aggregates with low GAG levels after 21 days of culture. Furthermore, no positive staining for collagen II was seen in MM under either of the culture conditions tested.

To find the optimal conditions for chondrogenesis, we cultured HNC spheroids in CDM with various cytokine supplementation. A growth factor with possibly the longest history of use in cartilage biology is TGF-β^16^. All three isoforms of TGF-β are present in the developing skeleton, particularly in the regions where primitive mesenchymal tissue undergoes condensation and cartilage formation^16^, which led to the use of TGF-β1^16^, and later TGF-β3^50^ to induce chondrogenesis in MSC aggregate cultures. Notably, the assembly of small MM aggregates with 2.5 × 10^4^ cells was only observed in CDM with TGF-β1 but not in the ChDif medium, highlighting the potency of TGF-β1 in CC aggregation. Furthermore, in experiments with different cytokine CDM, TGF-β1/3 produced the smallest spheroids with compact structures for both auricular and nasal constructs, further emphasizing the role of TGF-β in the condensation of chondrogenic cells.

Although TGF-β3 is not sufficient to induce chondrogenic differentiation in monolayer culture, it is considered more potent for chondrogenesis in mesenchymal aggregate cultures as compared to TGF-β1^50^. In our study, the two isoforms were very similar with respect to their effect on spheroid size, viability, and structure. However, we could not detect collagen II in auricular spheroids with TGF-β1 in one of the donors, whereas with TGF-β3 collagen II was present in all spheroids, possibly suggesting a higher potency of TGF-β3 in auricular CC and CPC aggregates.

CTGF is a downstream effector of TGF-β and is even selectively targeted for the treatment of pathological conditions that implicate TGF-β, such as fibrosis^51^. CTGF is a multifunctional cytokine, primarily known for inducing proliferation, aggregation, and ECM synthesis in chondrocytes^36^. In our study, both TGF-β isoforms and CTGF showed similarities with regard to spheroid viability and growth. Spheroids grown in CDM with either of these factors were typically the smallest among their type cultured in other CDM, had the highest SYTOX signal among all conditions, and showed little to no growth in auricular constructs or moderate shrinkage in the case of nasal spheroids.

Regardless of the observed overlap between the function of CTGF and TGF-β, histological analysis revealed substantial differences with regard to the influence of the two factors on spheroid structure, whereby CTGF showed more similarities to IGF-1. Both CTGF and IGF-1 produced the least dense spheroids. CTGF upregulates IGF-1 expression in chondrocytes and is thought to induce cartilage ECM synthesis at least partly in an IGF-1-dependent manner^37^, which may explain the similarities between their impact on HNC spheroids. Constructs grown in IGF-1-containing CDM with or without CTGF, however, showed a more loosely structured ECM than those grown with CTGF alone and were characterized by a low density of cells distributed in a sponge-like matrix. Interestingly, nasCPC spheroids were an exception and had a somewhat similar compact ECM structure in all conditions. Histological analysis showed that the large size of the spheroids was linked with the aerated ECM organization pattern of the IGF-1 spheroids, rather than being caused by the increase in cell numbers. However, the precise mechanism through which IGF-1 impacts the ECM structure of HNC spheroids is yet to be determined.

The structural pattern and hypocellularity of IGF-1-grown spheroids combined with their large size and sparse cell distribution were possibly the key reasons for the relatively low ATP staining seen in these constructs. IGF-1 has a significant structural homology with insulin and similarly to the latter, is known to stimulate glucose transport and oxidation in vitro^38,39^. The reduction of ATP in spheroids grown with IGF-1 was therefore unexpected. An experiment with monolayer culture revealed that IGF-1 increased ATP levels in individual cells, which was especially visible at earlier time points before cell confluence. While having more intense staining, cells in IGF-1 CDM were slower to reach confluence and formed a less dense layer than those grown in the ChDif medium, whereby fluorescence intensities in both conditions approximated each other at the expense of the increased cell density in the ChDif at later time points. This result further suggests that the sparse cell density rather than the actual decrease in ATP levels might be the primary cause of low ATP staining in IGF-1-grown spheroids. Moreover, the pattern of total vs. non-viable cell nuclei distribution by TUNEL labeling corresponded with the staining observed by ATP and SYTOX labels. Nevertheless, further experiments are needed to confirm directly that ATP levels are not reduced in a 3D setting.

The influence of IGF-1 was especially visible with aurCCs and, even more so aurCPCs both in 3D and in monolayer cultures. In 3D, auricular spheroids showed the most pronounced hypertrophy and the distinctive aerated spheroid structure, whereas in 2D they responded to IGF-1 treatment with the highest increase in ATP levels. Up to nine times higher IGF-1 expression levels have been reported in auricular CCs as compared to nasoseptal counterparts^35^, suggesting that auricular cells are exposed to higher IGF-1 through paracrine and autocrine signaling, which may explain the exaggerated effect of IGF-1 in auricular cells in our study. The differences in responsiveness to IGF-1 were also seen between aurCCs and aurCPCs in our study, suggesting that aurCPCs may express even more IGF-1 than aurCC. Additional mechanisms, such as higher expression of the IGF-1 receptor could also be implicated in the observed distinct cell response to IGF-1. Finally, the previously mentioned differences in the expression of matrix-degrading MMPs between nasal and auricular cells could additionally account for the seemingly reduced influence of IGF-1 on the ECM structure of nasal spheroids compared to the auricular counterparts.

The primary motive for using IGF-1 and CTGF in our experiments was to stimulate the production of elastin, which grants elastic cartilage the necessary flexibility and resistance to torsion. Although the expression of elastin is crucial for the engineered auricular cartilage, achieving it ex vivo is challenging, especially in chondrocytes obtained from hyaline cartilage, which is more readily available as a donor tissue source^52^. Both, CTGF^18^ and IGF-1^19^ have been reported to improve the production of elastin in scaffold-free auricular cartilage constructs. Consistently, in our study, CTGF and IGF-1 alone and in combination strongly stimulated elastin production in all cell types, including nasal CCs and CPCs. Distinctively from other conditions, in spheroids grown with CTGF and/or IGF-1, elastin was distributed evenly throughout the constructs, as opposed to being localized in the spheroid core and edges, as often seen with ChDif and TGF-β. Thus, our results confirm the potential of these two cytokines for the TE of elastic cartilage.

Somewhat contradicting the existing reports, CTGF and IGF-1 led to relatively low aggrecan synthesis in HNC spheroids from one of the donors, when used in isolation. CTGF has been associated with increased proteoglycan synthesis in rabbit auricular^18^ and articular^53^ chondrocytes and similar effects of IGF-1 have been reported in bovine articular chondrocytes^54,55^. However, this discrepancy can be attributed to the species and/or anatomical origin of chondrocytes used in our study. Furthermore, while the IHC results were repeatedly confirmed in individual samples, only two donors were tested in our study. More tests are therefore required to confirm that scarce or absent staining was not incidental and related to the particular experiment or donor. Notably, however, when combined, CTGF and IGF-1 induced a strong production of all cartilage ECM proteins tested, including aggrecan, in all cell types and both donors, highlighting the strong synergistic relationship between the two cytokines.

## Conclusions

Our results demonstrate a robust assembly of small HNC spheroids on ULA plates and highlight the crucial importance of cytokines in influencing the size, structure, and composition of ECM in scaffold-free TE constructs. While the commercially available ChDif medium overall facilitated a good maturation of HNC cartilage spheroids, the effectiveness of cytokines and their combinations in producing cartilage ECM emphasize the potential of tailoring the medium composition to obtain aggregates that match the target HNC cartilage more closely. Such an approach could facilitate the production of small HNC aggregates, which in the case of their successful fusion as shown in our study, could provide the much-needed donor graft material for reconstructive procedures.

However, structural insufficiencies or unbalanced ECM composition of constructs, combined with the different responses of cell types to the cytokines emphasize the need for further experiments. In our study, CDM containing TGF-β3 or a combination of IGF-1 and CTGF stimulated the production of all tested cartilage ECM factors in all spheroid types and donors. However, the distribution and quantity of ECM factors were not always sufficient in constructs grown with TGF-β3, whereas IGF-1 and CTGF combination failed to produce cartilage-like ECM architecture. Different combinations of these three factors could, however, yield the desired structure and composition of cartilage spheroids. Ultimately, optimal cytokine composition needs to be individually determined depending on the target HNC cartilage type and donor cell source. Furthermore, mechanical tests must be included for the final assessment of the most optimal constructs.

From a methodological perspective, our results caution against the interpretation of viability and proliferation using ATP-based dyes, particularly when using cytokines that might influence glycolysis, like IGF-1. Furthermore, our experiments emphasize the need for caution when interpreting total fluorescence microscopy results of 3D aggregates, keeping in mind the possible influence on the density and distribution of cells, as well as ECM architecture. While more challenging, analysis of 3D aggregates on a single cell level (e.g. with confocal laser scanning microscopy) is therefore strongly desirable when establishing cytokine composition for spheroid growth and maturation.

Finally, it is important to note that the current proof-of-concept study has some limitations that require careful interpretation of the results presented. Primarily, the methods of monitoring viability using standard fluorescence microscopy do not allow for precise localization or quantification of non-viable cells within the aggregate. In addition, we found that the ATP dye used in our study may have been influenced by the composition of the culture medium, highlighting the need for careful consideration in the choice of cell trackers for monitoring viable cells. Further limitations include the relatively small number of donors, variation in donor viability, and the lack of donor age and gender matching due to the restricted access to the patient data and anonymization requirements. While the consistency of the trends observed among donors in different experiments and a high number of technical replicates provide credibility to the results, follow-up studies with a sample pool of matched donors are necessary for further optimization of scaffold-free HNC cartilage constructs.

## Electronic supplementary material

Below is the link to the electronic supplementary material.

Supplementary Material 1

## Data Availability

The data supporting the findings of this study are available within this paper and the supplementary material. Raw data can be made available upon individual reasonable request, please contact Dr. Johann Kern at Johann.Kern@medma.uni-heidelberg.de.
