# Supplementary material for: Small spheroids for head and neck cartilage tissue engineering
Source: Sci Rep. 2024 Dec 30;14:32114. doi: 10.1038/s41598-024-83847-w (PMC11686322; doi:10.1038/s41598-024-83847-w)
Supplement: Supplementary file 1 — Supplementary Material 1 [file 41598_2024_83847_MOESM1_ESM.pdf]

Supplementary Figures

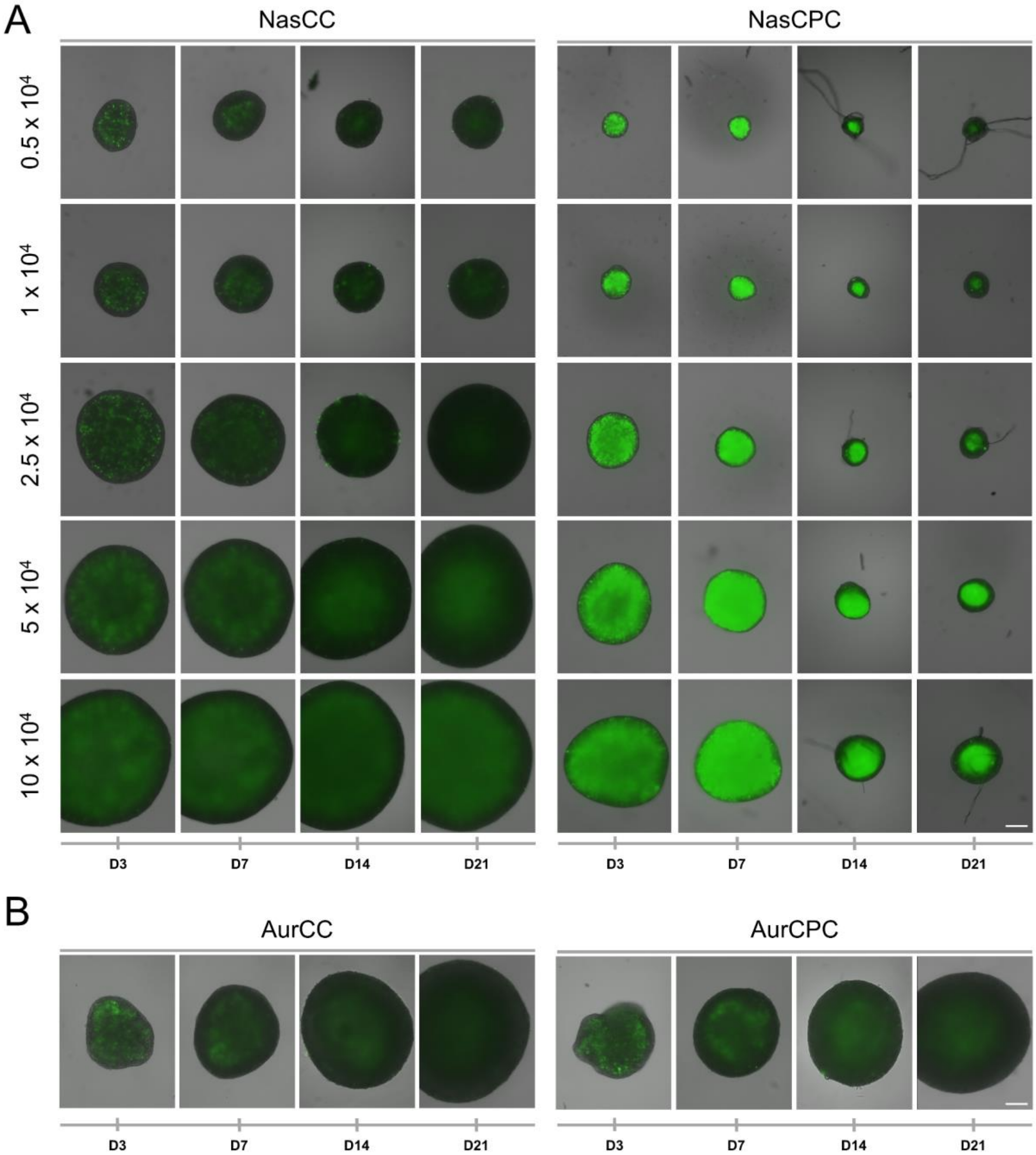

Figure S 1 Cell density and spheroid size. A. Nasal chondrocyte (CC) and chondroprogenitor (CPC) spheroids from donor 2 prepared with different cell densities after 21 days of culture in the StemMACS™ ChondroDiff (ChDif) medium. Micrographs depict an overlay of brightfield images and SYTOX™ Green fluorescent staining of non-viable cells. **B.** Auricular CC and CPC spheroids prepared from 1 x 10^4 cells. Scale bars 200 µm.

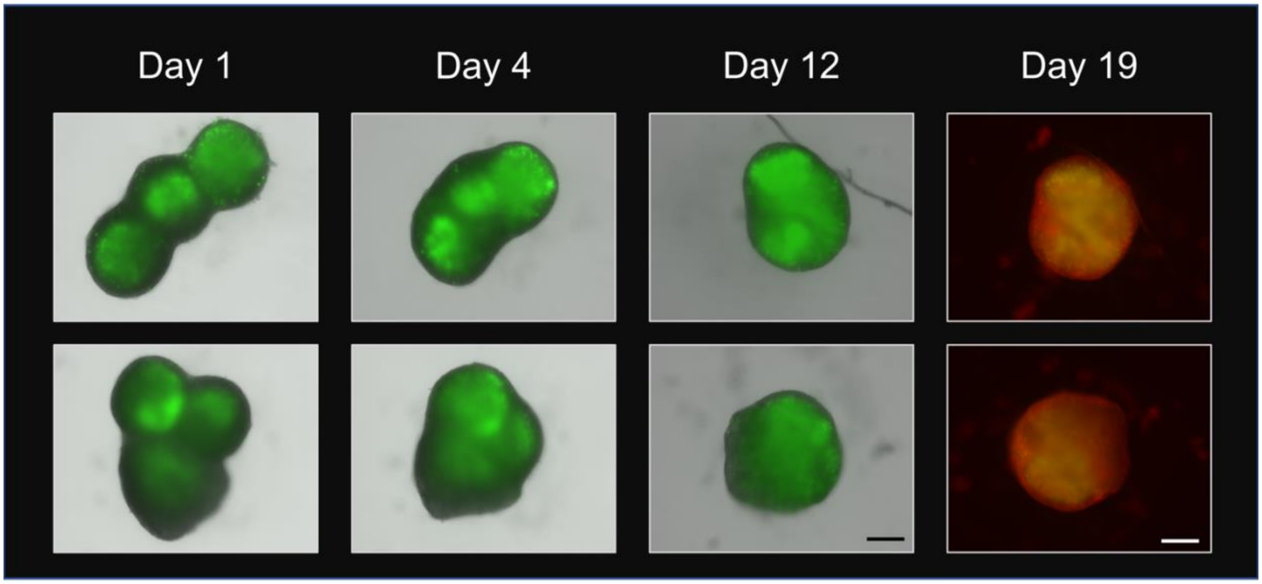

Figure S 2 Spheroid Fusion. Micrographs demonstrating a fusion of small spheroids prepared with 2.5 x10^4 cells. Green - SYTOX™ Green fluorescent staining of non-viable cells, Red - BioTracker ATP-Red Live Cell Dye. Scale bars 200 µm.

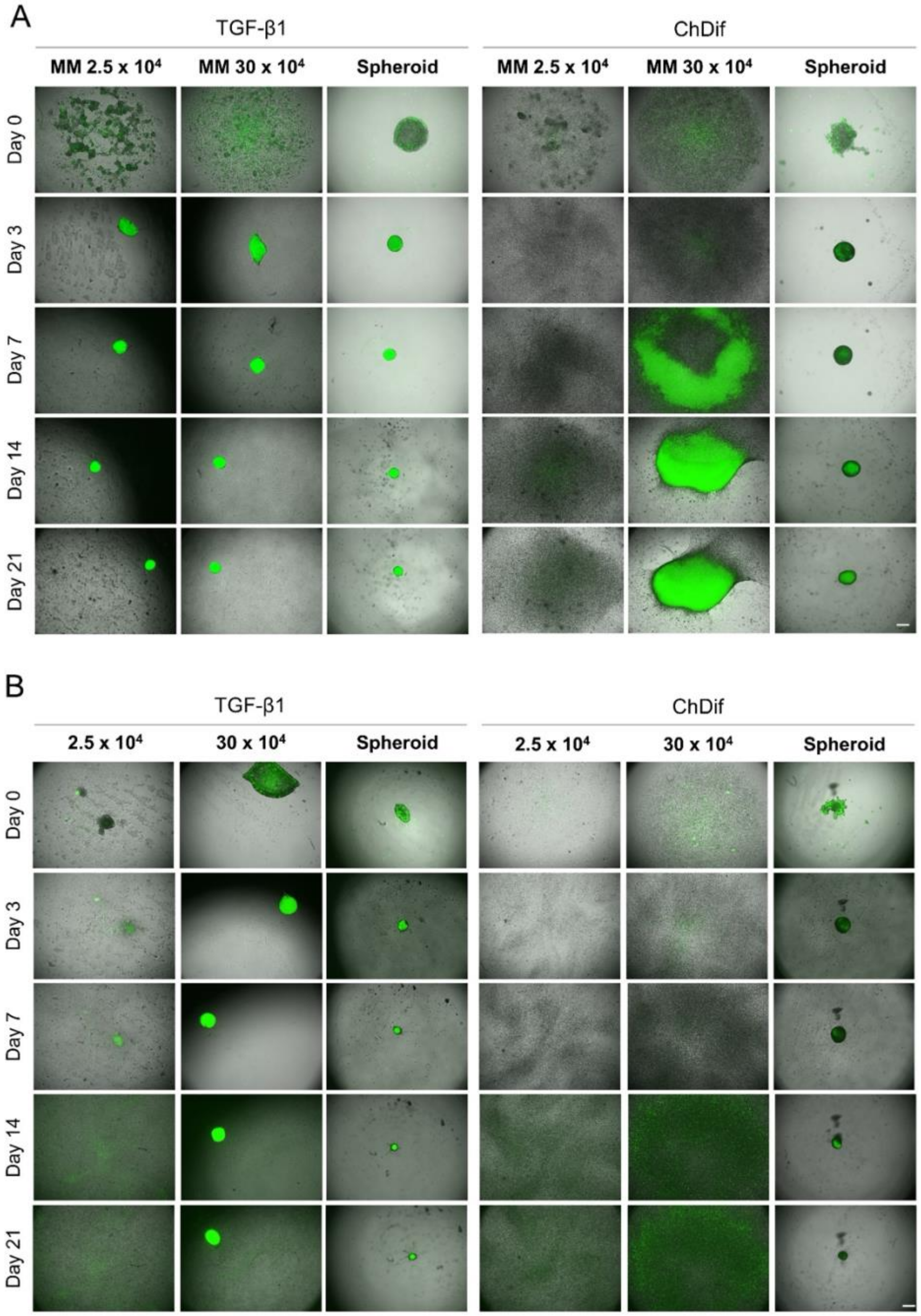

Figure S 3 Comparison of micromass and spheroid cultures. Viability staining of micromass (MM) aggregates prepared with two different densities of nasal chondrocytes (CC) and cultured in a standard medium supplemented with TGF-β1 or in StemMACS™ ChondroDiff (ChDif). Spheroids prepared from 2.5 x 10^4 cells were used for the reference. The culture was monitored for 21 days. No formation of MM cultures was observed in the ChDif medium for 2 out of 3 donors tested. Results for two donors (A and B) are shown. Scale bars 500 µm.

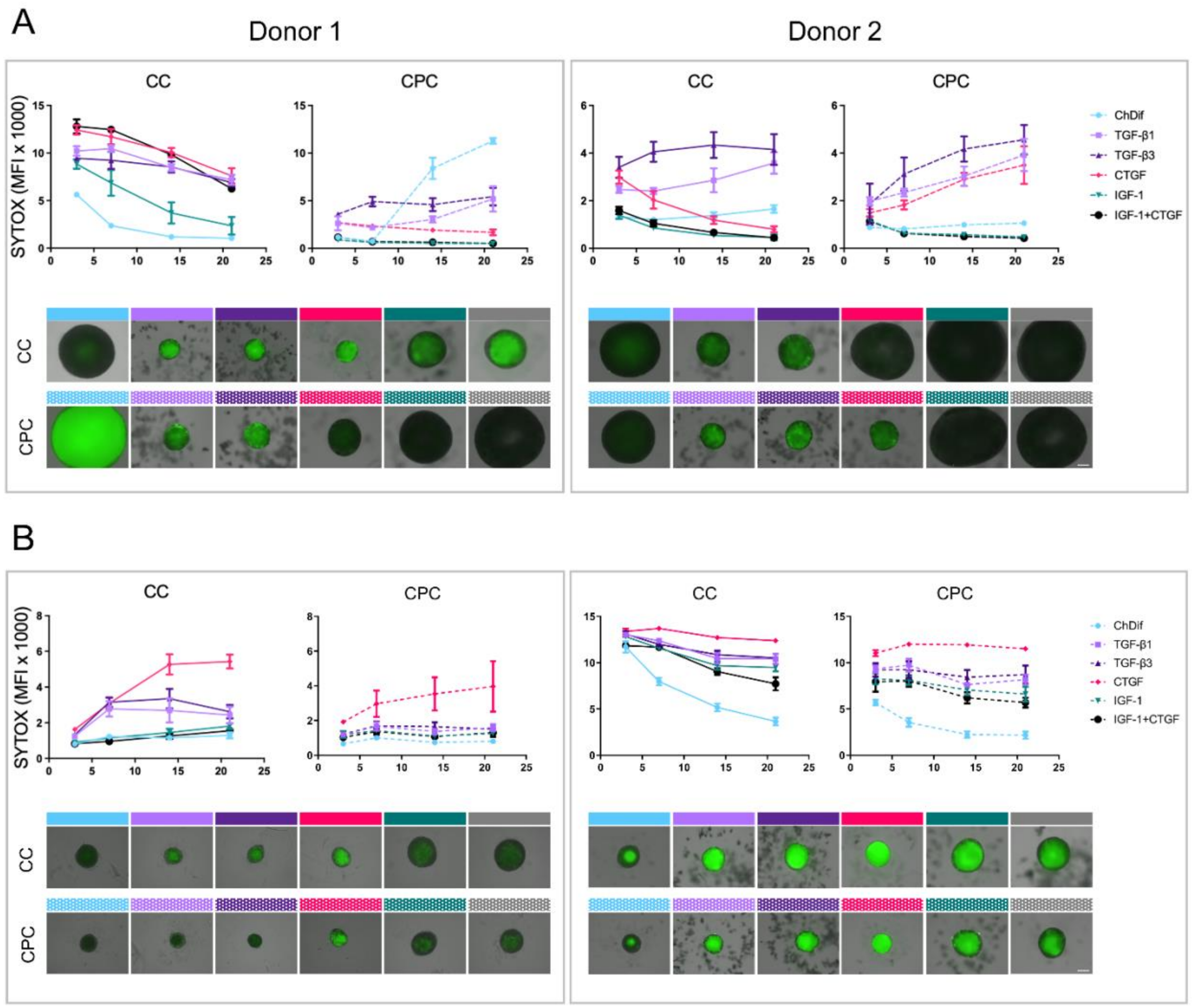

Figure S 4 The influence of cytokines on HNC spheroid growth, size, and viability. Mean fluorescence intensity (MFI) of SYTOX™ Green non-viable cell staining in **A.** auricular and **B.** nasal chondrocyte (CC, solid line) and chondroprogenitor (CPC, dashed line) spheroids from two different donors (donor 1 and 2). Results from culture days 3, 7, 14, and 21 in six different conditions (**Table 1**) are shown. Mean ± SD of 4 spheroids. Micrographs depict a representative spheroid on culture day 21 from each experiment. Scale bars 200 µm.

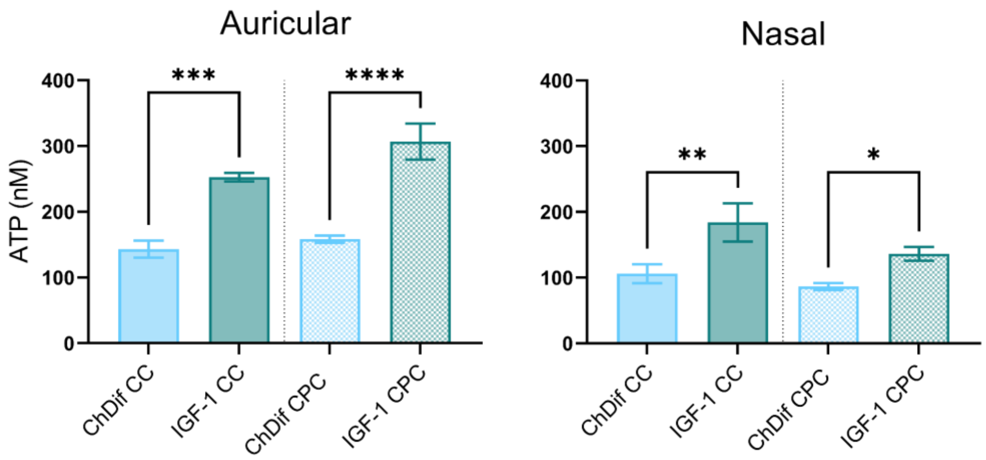

Figure S 5 The effect of IGF-1 on ATP. Total ATP concentration estimated by luminescence-based assay in auricular and nasal CCs and CPCs after 4 h in ChDif medium or chondrogenic differentiation medium (CDM) with IGF-1. Mean ± SD of 3 measurements, *p < .05, **p < .01, ***p < .001, ****p < .0001, one-way ANOVA.

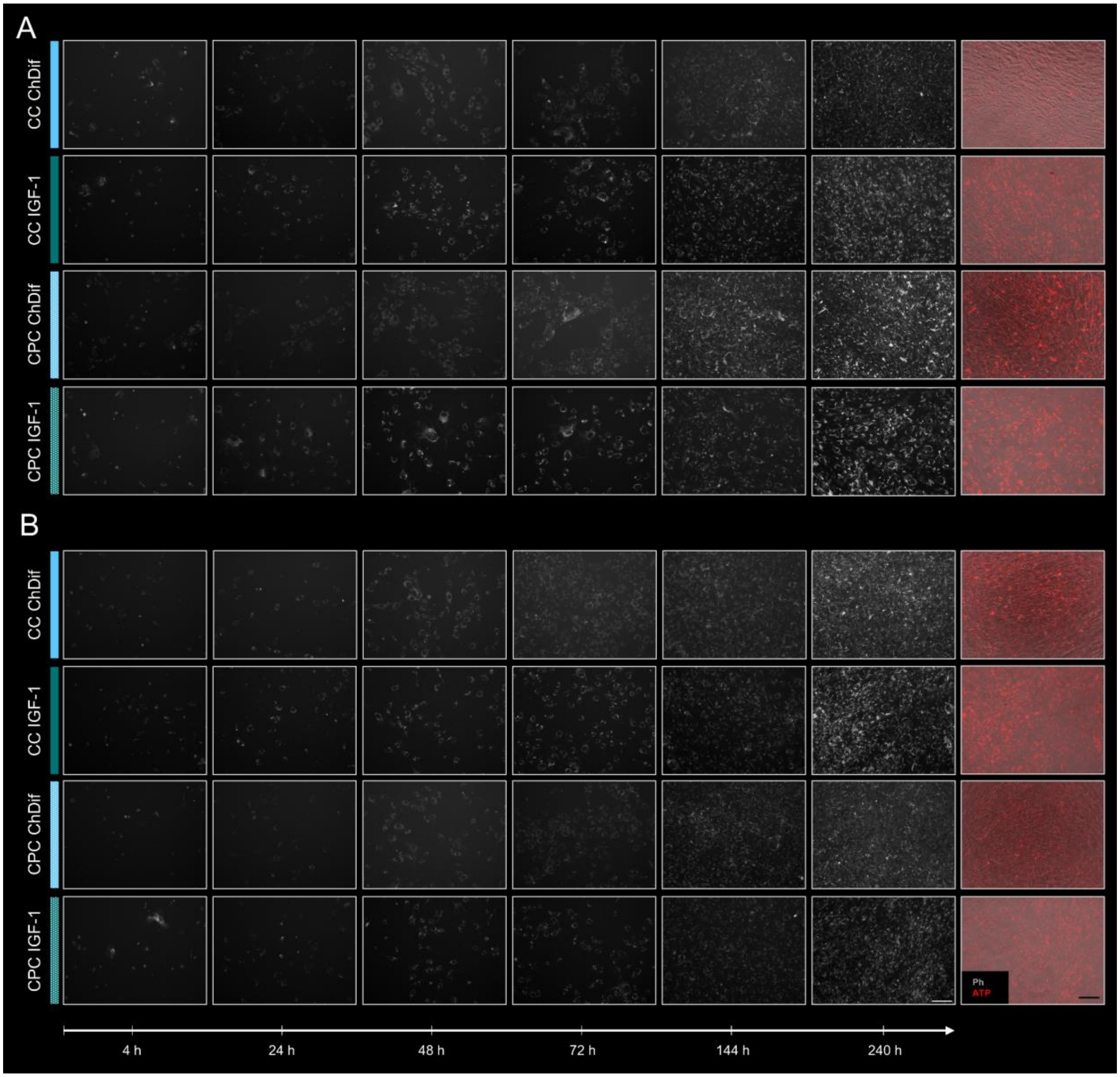

Figure S 6 The effect of IGF-1 on ATP. Micrographs depicting ATP fluorescence in (**A**) auricular and (**B**) nasal chondrocytes (CC) and chondroprogenitors (CPC) cultured in 2D in ChDif medium or chondrogenic differentiation medium with IGF-1 for up to 10 days (240 hours (h)). The final image depicts an overlay of phase contrast (Ph) and ATP at 240 h. Scale bar 100 µm.

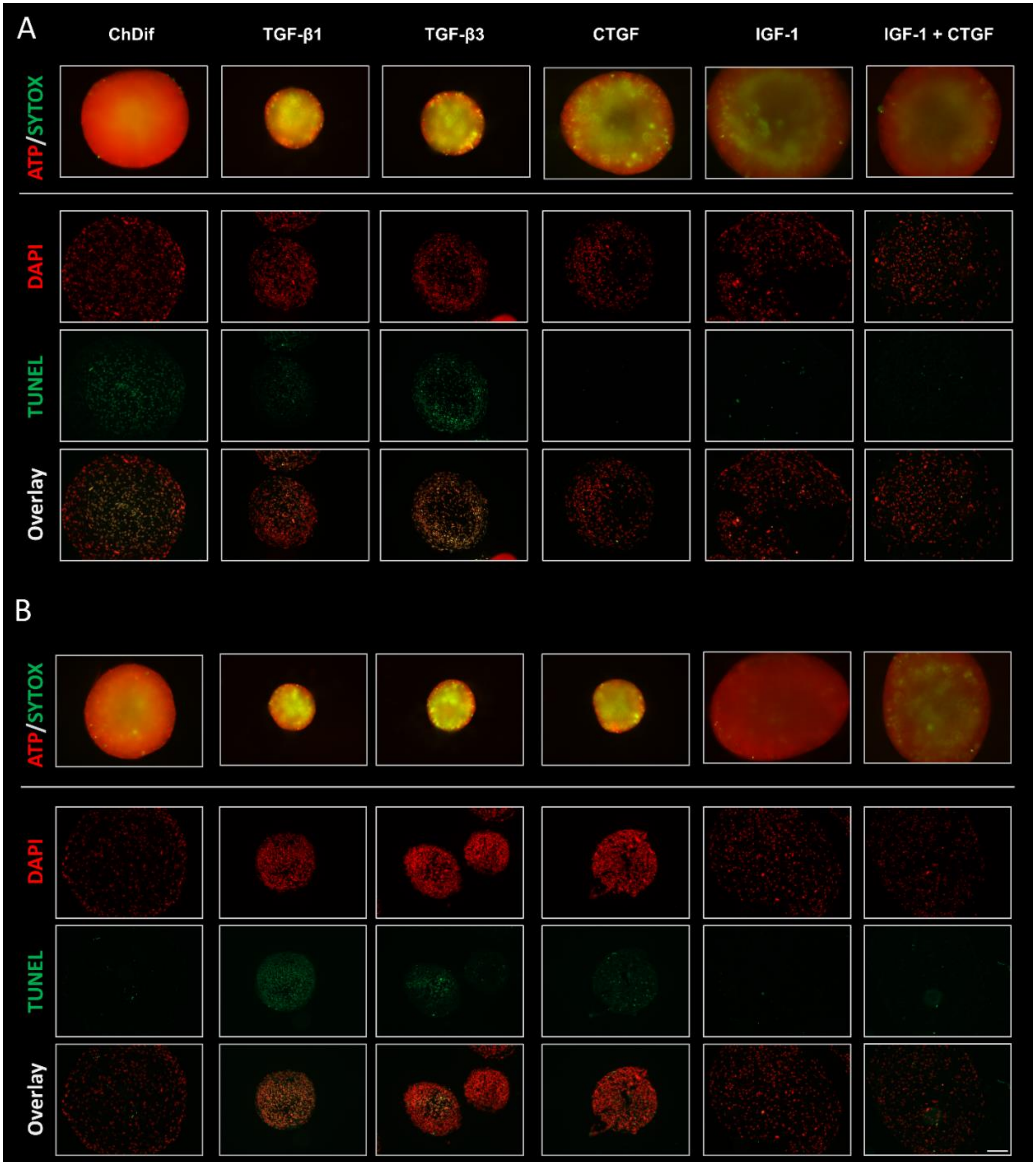

Figure S 7 Assessment of cell viability by TUNEL assay. A. The comparison of TUNEL and ATP/SYTOX viability staining patterns in auricular CC and **B.** CPC spheroids from Figure 3. Nuclear DAPI stain is false-colored in red, and TUNEL staining of fragmented DNA is shown in green. Scale bar 100 µm.

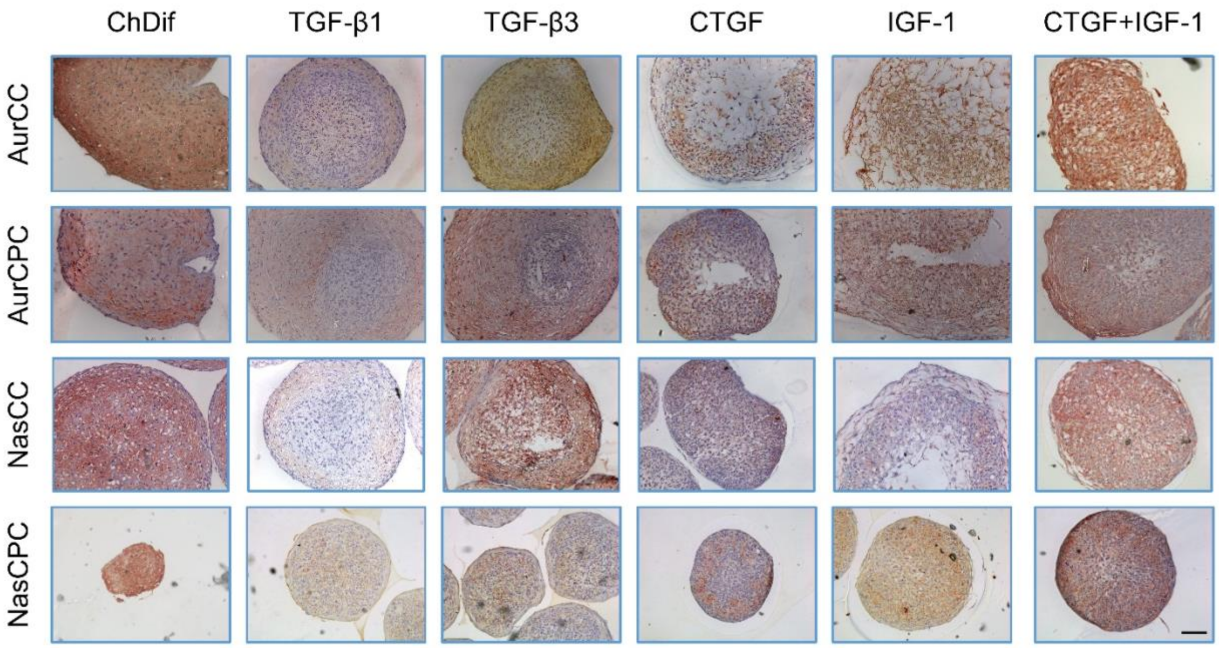

Figure S 8 Collagen I staining. Micrographs depict staining against collagen type I in auricular and nasal CC and CPC spheroids cultured in **(A)** ChDif or CDM containing **(B)** TGF-β1, **(C)** TGF-β3, **(D)** CTGF, **(E)** IGF-1, or **(F)** CTGF+IGF-1. The color of the micrograph border reflects the perceived intensity of the staining, as summarized in **(G)**. Scale bar 100 µm. AurCC/CPC - auricular chondrocytes/chondroprogenitors, nasCC/CPC - nasal chondrocytes/chondroprogenitors. ChDif - StemMACS™ ChondroDiff Medium, CDM - chondrogenic differentiation medium.

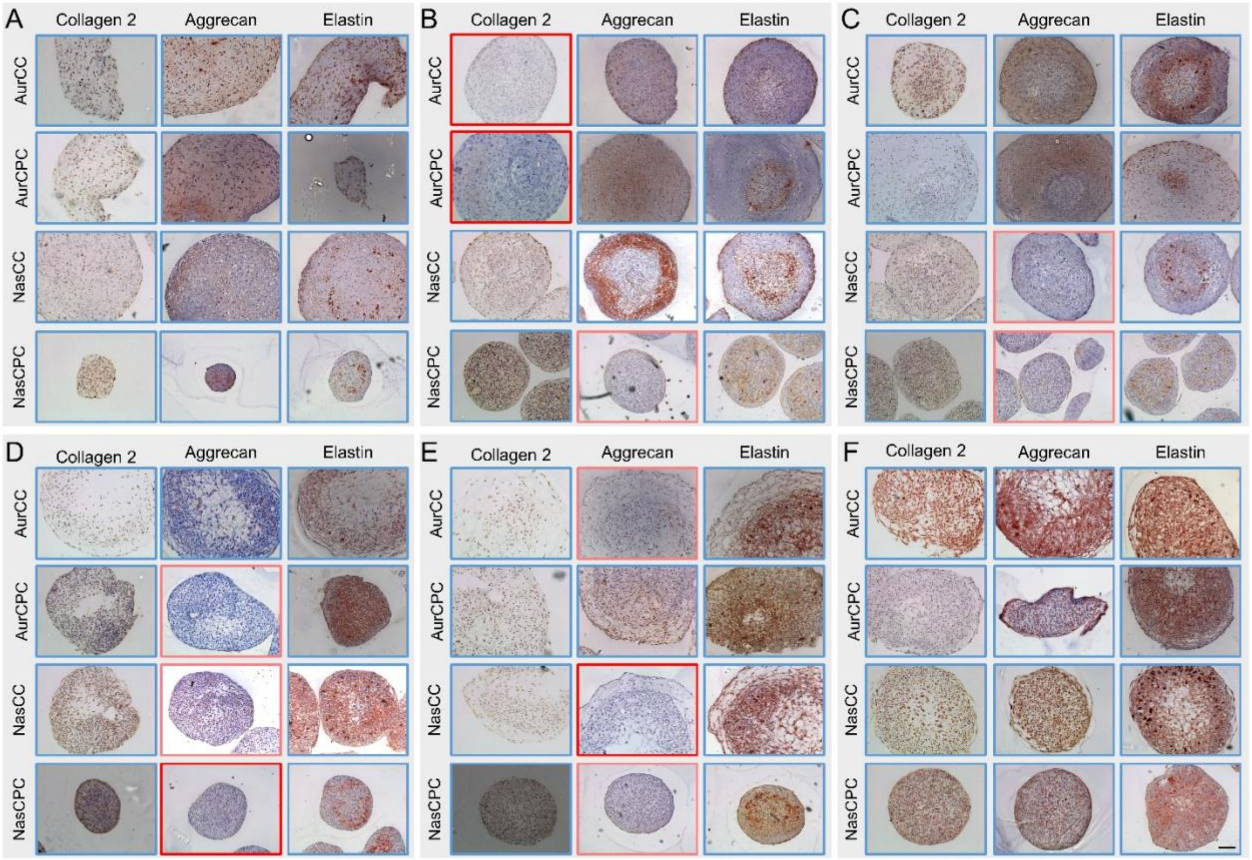

Figure S 9 Spheroid structure and cartilage ECM components. Micrographs depict the results of immunohistochemistry (IHC) for collagen type II, aggrecan, and elastic stain in auricular and nasal chondrocyte (CC) and chondroprogenitor (CPC) spheroids cultured in **A.** ChDif or chondrogenic differentiation medium (CDM) containing **B.** TGF-β1, **C.** TGF-β3, **D.** CTGF, **E**. IGF-1, or **F.** CTGF+IGF-1. The color of the micrograph border corresponds to the degree of staining, whereby positive staining is shown as blue, scarce/weak staining is shown as light red, and negative staining as dark red. Results from one of two independent experiments are shown. Scale bar 100 µm

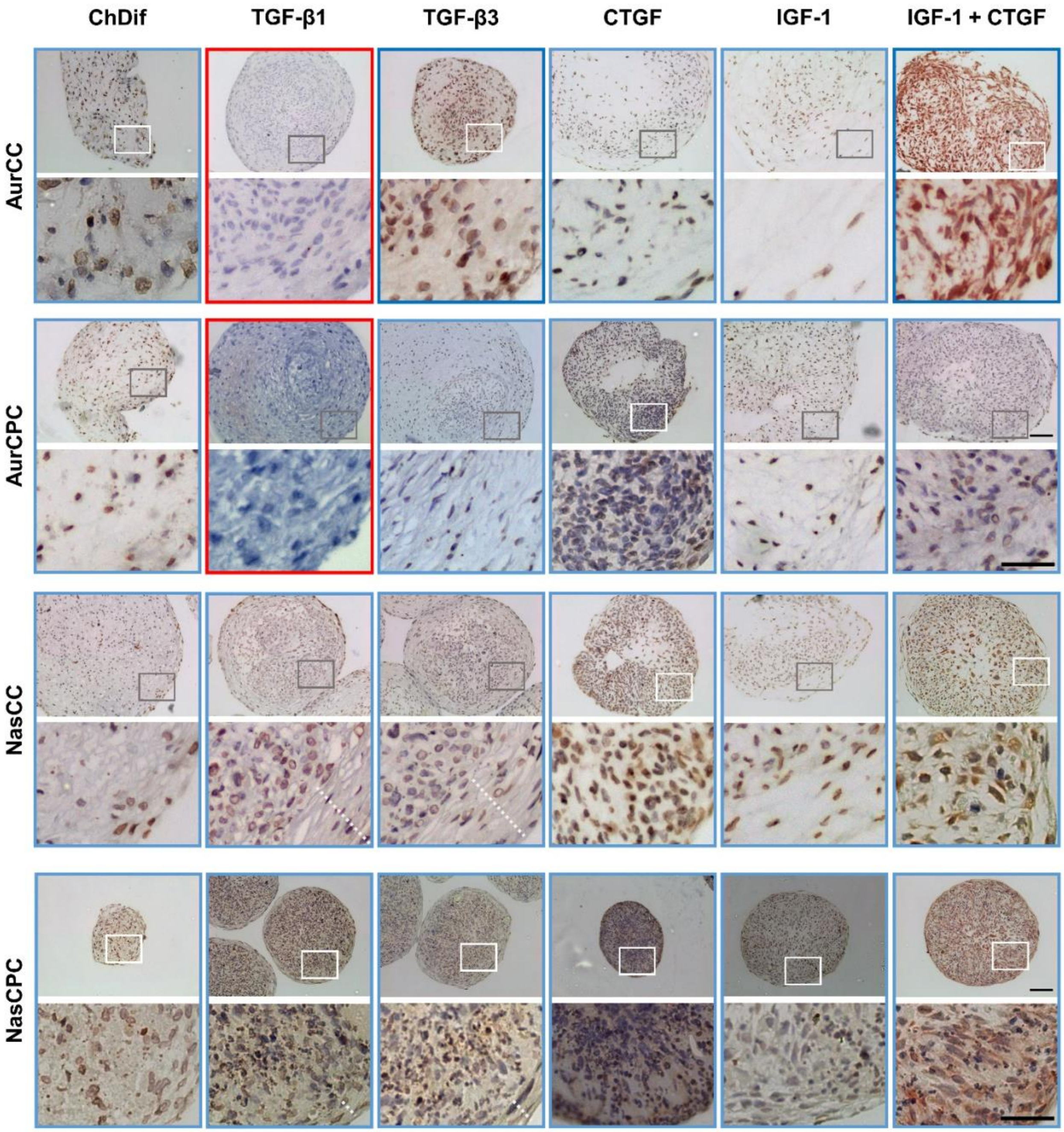

Figure S 10 Spheroid structure and collagen II staining. Micrographs depict auricular (aur) and nasal (nas) spheroids from chondrocytes (CC, top panel) and chondroprogenitors (CPC, bottom panel) stained against collagen type II. A magnified selection (marked rectangular region) is shown below each image. White dashed lines indicate a dense region of cells with parallel arrangement at the spheroid surface. The color code in the micrograph border reflects the positivity of the staining, marked in blue, or red when staining is absent. Scale bars 100 µm (top) and 50 µm (bottom).
